# From bites to ripple effects: Unraveling the health, economic, and social effects of arboviral epidemics in Mainland France

**DOI:** 10.1016/j.ijregi.2026.100922

**Published:** 2026-05-25

**Authors:** Bénédicte Apouey, Véronique Raimond, Elodie Rouvière, Carine Milcent, David Roiz, Jean-Michel Salles, Frédéric Simard, Yannick Simonin, Josselin Thuilliez, Marie-Claire Paty

**Affiliations:** 1 National Centre for Scientific Research (CNRS), Paris, France; 2 ANSES (French Agency for Food, Environmental and Occupational Health & Safety), Maisons-Alfort, France; 3 SADAPT, Univ. Paris-Saclay, AgroParisTech, INRAE, Palaiseau, France; 4 Paris School of Economics-CNRS, Paris, France; 5 MIVEGEC, Univ. Montpellier, IRD, CNRS, Montpellier, France; 6 CEE-M, CNRS, INRAE, Institut Agro, Univ. Montpellier, Montpellier, France; 7 Univ. Montpellier, Montpellier, France; 8 Santé Publique France, Saint-Maurice, France; 9 CNRS, Rennes, France

**Keywords:** *Aedes albopictus*, Dengue, Chikungunya, Zika, Socioeconomic effects, Mainland France

## Abstract

**Objectives:**

Arboviral diseases are posing an increasing burden in temperate regions. This article documents the potential effects of outbreaks and epidemics caused by an arbovirus transmitted by *Aedes albopictus*, across multiple domains, in mainland France, which has been minimally affected by such outbreaks so far.

**Methods:**

We draw on the knowledge accumulated in mainland French regions that have dealt with limited autochthonous outbreaks, and in the overseas departments and regions of France which are regularly affected by epidemics. We present an original method that combines a systematic review of the literature, the analysis of qualitative data from questionnaires completed by stakeholders, and our expert input.

**Results:**

We identify several key vulnerabilities such as healthcare strain, economic losses, and social disruption. Our analyses highlight specific risks and preparedness gaps for temperate areas.

**Conclusion:**

We underscore the critical importance of allocating sufficient resources to vector management and of developing intersectoral policies to mitigate the effects of future epidemics across several domains.

## Highlights

• Autochthonous chikungunya and dengue cases are sharply increasing in mainland France.
• We document potential effects of *Aedes albopictus*-associated outbreaks/epidemics.
• Main risks include healthcare strain, economic losses, and social disruption.
• We emphasize intersectoral action and resource allocation for vector control.

## Introduction

Arboviruses have been expanding their geographic range throughout the world because of climate change and increased global travel and trade. In Europe, *Aedes albopictus* is now widespread and has been implicated in autochthonous transmission of dengue virus (DENV), Zika virus (ZIKV), and chikungunya virus (CHIKV). From 2010 to 2023, almost 300 cases of autochthonous dengue were reported in Europe.^1^ In 2024, more than 300 cases were reported, mainly in Italy (213). Regarding chikungunya, more than 200 cases were reported in 2007 in north-eastern Italy and more than 400 cases in 2017 in the Lazio and Calabria regions of Italy.

**Figure 1:**
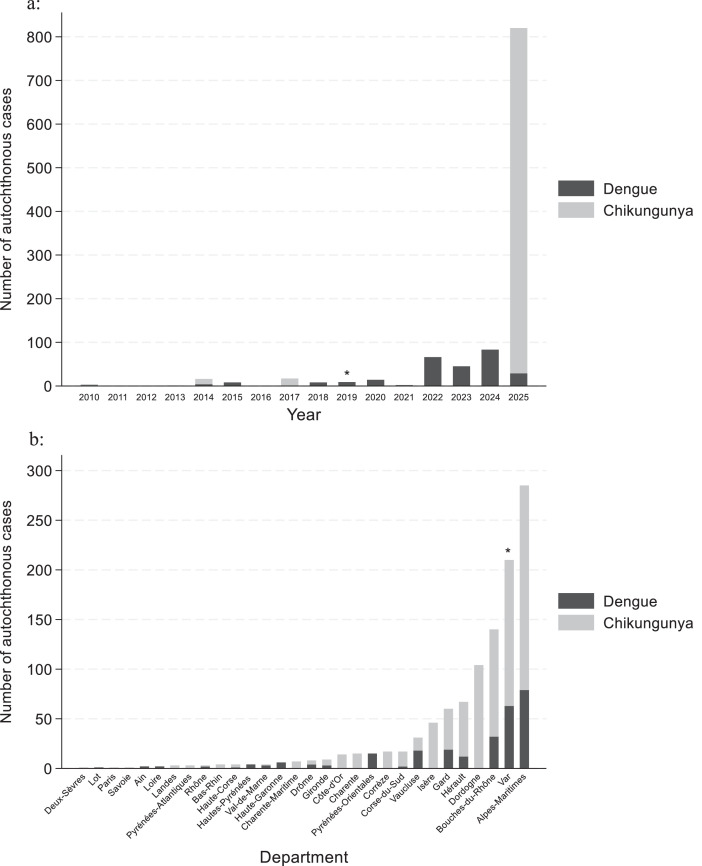
Number of autochthonous cases of dengue, chikungunya, and Zika in mainland France, 2010-2025. a. Annual number of autochthonous cases in mainland France, 2010-2025. b. Number of autochthonous cases by department in mainland France, 2010-2025. * In 2019, three autochthonous cases of Zika were reported in the Var department. Sources : Santé publique France website. https://www.santepubliquefrance.fr/maladies-et-traumatismes/maladies-a-transmission-vectorielle/chikungunya/documents/article/emergences-de-dengue-et-de-chikungunya-en-france-metropolitaine-2010-2018https://www.santepubliquefrance.fr/maladies-et-traumatismes/maladies-a-transmission-vectorielle/chikungunya/documents/article/emergences-de-dengue-et-de-chikungunya-en-france-metropolitaine-2010-2018. https://www.google.com/url?sa=t&source=web&rct=j&opi=89978449&url=https://www.santepubliquefrance.fr/media/files/01-maladies-et-traumatismes/maladies-a-transmission-vectorielle/chikungunya/synthese-des-episodes-de-transmission-autochtone-de-chikungunya-dengue-et-zika&ved=2ahUKEwjxgM_O_JeSAxXVWaQEHcHvNckQFnoECBkQAQ&usg=AOvVaw2FfbKixAm4j0jlxKvsBNld https://www.santepubliquefrance.fr/maladies-et-traumatismes/maladies-a-transmission-vectorielle/chikungunya/articles/donnees-en-france-metropolitaine/chikungunya-dengue-et-zika-donnees-de-la-surveillance-renforcee-en-france-metropolitaine-en-2023 https://www.santepubliquefrance.fr/content/download/773451/4873882?version=1

This paper focuses on mainland France, where *Ae. albopictus* was first detected in 2004 in the Alpes-Maritimes department.^2^ Since then, colonization has steadily progressed, and by early 2025, *Ae. albopictus* had spread to 81 departments across mainland France. Autochthonous transmission events of dengue and chikungunya have been observed since 2010 (Figure 1). Sixty-five autochthonous cases of dengue were reported in 2022,^3^ 45 in 2023,^4^ and 83 in 2024^5^ [1]. Three chikungunya outbreaks^6^ occurred in mainland France in 2010, 2014, and 2017 [1,2]. In 2024, an autochthonous case of chikungunya was identified in the Paris region. In 2025, 834 autochthonous chikungunya and dengue cases were detected in mainland France.^7^ In addition, three autochthonous Zika cases were reported in 2019 in the Var department—marking the first autochthonous Zika cases acquired through vector-borne transmission in the European Union/European Economic Area.^8^

In a recent report by the French Agency for Food, Environmental and Occupational Health and Safety (ANSES) [3], to which we contributed, it is emphasized that the probability of an epidemic in mainland France is relatively high.^9^ However, knowledge and data on the consequences of such outbreaks/epidemics in temperate zones remain limited, although this information is essential to develop policies that mitigate the effects of outbreaks/epidemics.

This article explores the potential effects of an outbreak/epidemic caused by an arbovirus (specifically DENV, CHIKV, and ZIKV), transmitted by *Ae. albopictus*, in mainland France, which has been minimally affected by outbreaks so far, but is experiencing an upward trend in autochthonous transmission. We identify potential effects across multiple domains and discuss the affected populations, time frame, and level of DENV, CHIKV, or ZIKV transmission at which the effects are expected to emerge, to the extent possible. Because arboviral outbreaks/epidemics may generate cascading impacts across institutional, health, economic, and social domains, a single-sector analysis would fail to capture their full consequences that should shape preparedness and response. We also examine the relationships between the effects we identify, as well as feedback loops. We leverage the multidisciplinary expertise of our team—economics, epidemiology, public health, ecology, entomology, virology—to analyze these potential effects in multiple domains and the links between them.

Given the lack of data on the effects of epidemics in comparable areas, this paper draws particularly on the knowledge accumulated in the overseas departments and regions of France (DROMs), which are regularly affected by arboviral epidemics, and in the French mainland regions that have dealt with clusters of autochthonous transmission. We develop an original framework that combines a systematic literature review, an opportunistic search, qualitative material (from questionnaires administered to stakeholders in France), and our expert input.

**Figure 2:**
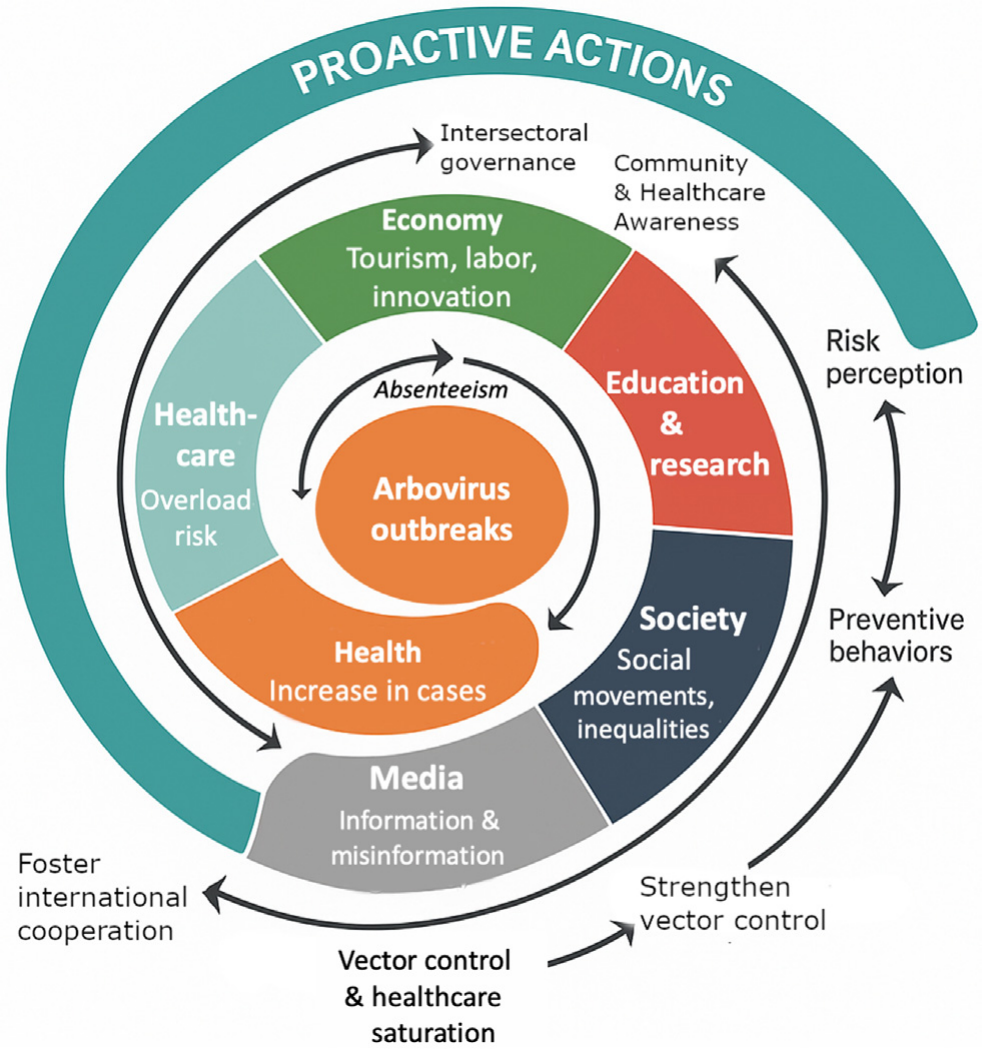
Overview of the effects in multiple domains.

After presenting our method, the reported impacts are grouped into several operational domains for which (positive or negative) impacts can be assessed and documented, their underlying mechanisms can be unraveled, and relevant stakeholders can be identified for preparedness and action. Figure 2 provides an initial overview of the domains in which impacts of an outbreak/epidemic are identified.

## Method

This paper particularly relies on data from the DROMs,^10^ focusing on Guadeloupe and Martinique in the Caribbean (Lesser Antilles), French Guiana in South America, and Réunion in the Indian Ocean. DROMs function under the same administrative and decision-making systems as mainland France. The Social Security framework, including health insurance, is identical in mainland France and the DROMs, based on the principles of universal healthcare. Governance is also centralized: the Ministry of Health oversees both mainland France and the DROMs, through Regional Health Agencies (RHAs, “Agences régionales de santé”) present in each region.

In using knowledge gained from the DROMs, we take into account several key differences between these territories and mainland France. Whereas mainland France experiences a temperate climate, DROMs are located in tropical^11^ or equatorial^12^ climate zones. In mainland France, *Ae. aegypti* is not established and *Ae. albopictus* is the only competent vector. In contrast, in French Guiana, Martinique, and Guadeloupe, *Ae. aegypti* is the principal vector, while in Réunion, *Ae. albopictus* is the main vector but *Ae. aegypti* is also present. In mainland France, mosquitoes enter winter diapause that interrupts vector cycles and limits the epidemic dynamics over long periods, in contrast with tropical and equatorial areas. Dengue is not endemic in mainland France (and in mainland European Union/European Economic Area), while dengue outbreaks/epidemics are frequently reported in French Guiana, Guadeloupe, Martinique, and Réunion. Therefore, the population in mainland France is immunologically naive to *Aedes*-borne diseases, in contrast with the DROMs. Moreover, DROMs face specific health challenges, with a higher prevalence of conditions such as diabetes,^13^ obesity,^14^ and sickle cell disease,^15^ which may influence individual responses to arboviral infections. Finally, compared to mainland France, healthcare access and service provision are characterized, in the DROMs, by more pronounced shortages of healthcare professionals (particularly in French Guiana and Mayotte) and greater inequalities in access to specialized care and advanced medical equipment.

Given the lack of comprehensive assessments on the multisectoral effects of an outbreak/epidemic (due to an arbovirus transmitted by *Ae. albopictus*) in Europe or in temperate regions, and because evidence from equatorial and tropical settings is not directly transferable to mainland France, we develop an original framework grounded in methodological triangulation. We combine a systematic literature review, an opportunistic search, qualitative material, and our expert input (derived from discussions within our multidisciplinary expert team, and our own professional and research experience). The systematic literature review, shaped by specific criteria and highly focused in scope, employs the PICO method (Patient/population, Intervention, Comparison, Outcomes). The opportunistic search yields additional academic articles, as well as grey literature (institutional reports and newspaper articles). Qualitative data come from responses to questionnaires administered to various field stakeholders in mainland France and the DROMs in 2024: RHAs, regional mosquito control operators, clinicians, academic hospitals managers, the French Blood Agency, and Chambers of Commerce and Industry. More details about the method are provided in Supplement A and some respondents’ verbatim quotations are reported in Supplement B.

## Prevention, control, and management

In the event of an epidemic, the first actors to be affected would be those involved in the prevention, control, and management system. This section presents this system in mainland France under routine conditions and in the context of recent challenges (arising from the 2022-2024 surge in autochthonous cases), and also considers, from a prospective perspective, what would occur in the event of an epidemic.

The surveillance of arboviral diseases transmitted by *Ae. albopictus* mainly relies on mandatory declaration^16^ of human cases by health professionals to RHAs and the National Public Health Agency.^17^ Surveillance operates year-round and is strengthened from May to November—the period of vector activity—through increased awareness among healthcare professionals and automated lab result reporting, in addition to mandatory declaration.

A mandatory declaration to a RHA during the period of vector activity triggers an epidemiological and entomological investigation: when a case is identified, the RHA and the National Public Health Agency conduct a door-to-door investigation to locate mosquito breeding sites, identify other cases, and promote the adoption of preventive behaviors.

Vector control interventions are readily implemented in the area(s) where the infected individual(s) have been staying during the previous days, as an emergency countermeasure to prevent further virus transmission. These interventions include public awareness on vector-borne disease prevention, elimination of larval breeding sites, larvicidal treatments, and adulticidal control to reduce the longevity and density of potentially infected female vectors.

RHAs delegate their vector control activities to local mosquito control operators. Whenever new autochthonous cases are declared, agents in charge of vector surveillance and control thus serve as the first lines of defense. Because of the unusually high number of imported and autochthonous cases since 2022, the workload of RHAs and operators has been increasing. In their responses to our questionnaires, RHAs from various regions across mainland France reported facing a significant risk of saturation, as human, material, and financial resources were not increasing at the same pace as needs (Supplement B, Table B.1, rows D1-1 to D1-5). The situation was particularly tense in regions not used to facing mosquito-borne diseases. Staff members working in RHAs had no dedicated resources and had, therefore, to monitor several dengue cases while managing other health priorities. In regions more familiar with the risk, mainly in the southeast of France and in Corse, local operators reported a risk of lower-quality field interventions, because of excessive workload and worsening working conditions (Supplement B, Table B.1, row D1-6).

In case of an epidemic, we expect RHAs to scale-up social mobilization initiatives (Supplement B, Table B.1, row D1-7). Note that a major outbreak would trigger the Arboviral Crisis Plan.^18^

Should surveillance, management, and vector control activities become overwhelmed, the outbreak may rapidly escalate beyond control, triggering ripple effects across multiple domains. These effects may manifest at different levels of transmission (Supplement C), according to our expert team. Table 1 reports the key effects identified across various domains, as well as the levels of transmission at which they may occur.

**Table 1:** Summary of the main effects according to transmission intensity levels.

	Intensity levels of autochthonous transmission at which effects emerge^a^	Key effects
**Prevention, control and management**	1	• Risk of saturation of Regional Health Agencies and mosquito control operators. • Social mobilization initiatives.
**Population health**	1	• Severe or chronic complications (e.g. Guillain-Barré syndrome, long-term joint pain) disproportionately affecting vulnerable groups (e.g. the elderly, children, pregnant women).
**Healthcare**	2	• Risk of hospital and emergency service saturation during summer outbreaks/epidemics, because of high case numbers and staff shortages.
**Economic activity**	2	• Productivity losses because of absenteeism per infection, especially in construction and outdoor sectors. • Trip cancellations in highly touristic regions, rerouting.
**Education**	3	• Missed school days (from child or teacher illness and school closures). • Long-term impacts on cognitive development.
**Research**	2	• Funding surges for vaccines, diagnostics, and vector control, but often fading support after the crisis.
**Society**	2	• Amplified exposure and vulnerability because of poor housing, lack of air-conditioning, and social isolation.
**Information and misinformation**	2	• Influence on behaviors and outbreak/epidemic control. • Amplification or mitigation of the outbreak/epidemic and its effects.

Effects presented in this table are identified through a literature review, qualitative material, and the authors’ input.

a Transmission levels: level 1 = episode of autochthonous transmission; level 2 = localized outbreaks; level 3 = epidemic; level 4 = major epidemic.

## Population health and healthcare

Arboviral outbreaks caused by DENV, ZIKV, and CHIKV may significantly affect public health (from transmission level 1 onward) and the healthcare system (from level 2 onward) in mainland France.

Given the limited prior exposure, the majority of the population in mainland France is immunologically naive to these viruses. Therefore, an initial outbreak would likely lead to a higher proportion of symptomatic infections compared to endemic regions. In the case of dengue however, the absence of previous exposure to the virus may limit the occurrence of severe dengue symptoms, as typically observed in secondary infections with different serotypes.

Health impacts are influenced by the incidence of severe or chronic cases and long-term complications, such as neurological sequelae (e.g. Guillain-Barré syndrome or chronic cognitive impairment). Vulnerable groups—including children, the elderly, pregnant women, immunocompromised individuals, those with comorbidities (such as high blood pressure and diabetes), and, for dengue, patients with severe sickle cell syndrome—are at a higher risk of severe outcomes, as reported by stakeholders from the DROMs (e.g. Supplement B, Table B.1, row D2-1) and supported by clinical guidance.^19^

The healthcare system in mainland France is, in principle, better capable of providing high-quality patient care than that of many low- and middle-income countries affected by arboviral epidemics. However, according to our expert team, (pre-)existing weaknesses related to access to healthcare may exacerbate the detrimental effect of an epidemic on population health. First, mainland France is experiencing an expansion of medical deserts which are associated with delayed diagnosis and treatment for primary care services. Second, the risk of saturation, particularly in emergency services, usually increases during the summer period, which is the primary season for outbreaks. This time of the year is marked by reduced staffing because of holidays, which directly affects the system’s ability to provide timely care.

Epidemics worsen the risk of saturation because of an increase in the demand for care (related to arboviral cases) combined with staff shortage (caused by arboviral illness among healthcare professionals), as observed in the DROMs by a clinician (Supplement B, Table B.1, row D2-1). Our expert team considers that this logic would also apply to mainland France and may even be more pronounced, as the mosquito season coincides with the summer holiday period and reduced availability of healthcare professionals.

The healthcare system saturation may exacerbate the direct negative effect of an epidemic on public health by (i) deteriorating the health outcomes of arboviral patients because of delayed or lower-quality care, (ii) increasing viral transmission because of delays or failures in the detection and isolation of cases, and (iii) worsening care for other health conditions.

The effects of an outbreak on population health may go beyond the healthcare sector and trigger ripple effects across other domains.

## Economic activity

According to several stakeholders, epidemics may generate non-negligible economic disruptions in mainland France,^20^ although their magnitude remains highly uncertain (Supplement B, Table B.1, rows D3-1 to D3-3).^21^ We expect economic effects to emerge from transmission intensity level 2 onward. Economic effects are expected to take several forms: effects of absenteeism, impact on the tourism sector, adaptive behaviors by firms, and effects on health-related markets (e.g. vaccines). We examine each in turn.

One channel through which outbreaks/epidemics would affect economic activity is work absences, that may generate losses in labor productivity (Supplement B, Table B.1, rows D3-2 and D3-3)^22^ and global supply chain disruption [7]. Work days lost depend on the disease and its severity. Some estimates on work days lost from the literature appear relevant for mainland France. In particular, in a global study on dengue [8], the number of work days lost is assumed to be 4.2 (dengue fever, ambulatory), 9.9 (dengue fever, hospitalized), and 14 (severe dengue, hospitalized). For the 2014-2015 chikungunya outbreak in the US Virgin Islands, employed case-patients missed 9 days in the 12 months following their disease onset [9]. Sectors characterized by higher occupational exposure to mosquitoes (e.g. construction) are likely to be disproportionately affected.

The tourism sector would be one of the first to be affected by an epidemic, although few quantitative studies accurately assess the magnitude of this effect [10,11]. Both the literature and the tourism stakeholder testimonies acknowledge that epidemics in highly touristic areas disproportionately affect local professionals and that this effect might be particularly significant for France, given its status as a popular tourist destination [11] (Supplement B, Table B.1, rows D3-1 and D3-4). Our expert team agrees with this assessment. Well-informed travelers are more likely to cancel trips [12,13], potentially rerouting tourist flows to safer regions—a phenomenon warranting further research.

Individuals and businesses are likely to adopt or implement preventive measures (such as using repellents and fly screens and wearing long sleeves) to avoid bites and thus mitigate potential income or revenue losses. Available studies show that prevention practices in the tourism sector tend to increase [14]. In addition, to limit productivity losses because of absenteeism, business managers are often willing to absorb the additional prevention-related costs [15]. While such measures may entail short-term cost increases, they would be offset by reduced economic burden and also contribute to the control of the outbreak/epidemic [16].

More generally, during outbreaks and epidemics, private and public agents often adapt their behavior to avoid contamination or dissemination of the disease. Our expert team expects these preventive behaviors to contribute to the growth of markets for mosquito bite personal protection, vaccines, treatments, and insurance products, thereby stimulating innovation (as indicated by Chambers of Commerce and Industries, Supplement B, Table B.1, row D3-5).

## Education and research

Effects on education, which are expected to be observed from transmission intensity level 3 onward, can be conceptualized in terms of two dimensions. The first relates to missed school days by children because of their own illness or to school closures, teacher absences, or parents’ inability to bring their children to school (because of their own illness or caregiving responsibilities) [17]. Cases of school absenteeism are reported in the questionnaires by RHAs from the DROMs, including for teachers (Supplement B, Table B.1, rows D4-1 to D4-3).

The second dimension pertains to the impact of child illness on learning outcomes and cognitive development (e.g. van Aalst *et al.* [18] on chikungunya). However, there is no country-wide quantification of these effects, to the best of our knowledge. Exploring the relationship between arboviral diseases and academic performance is an important open area of research (Supplement B, Table B.1, row D4-1).

Understanding the educational effect is just one facet of the broader consequences arboviral outbreaks/epidemics impose on the population. From transmission intensity level 2 onward, outbreaks may also critically affect research dynamics, funding, and innovation—areas that significantly shape our preparedness and response capabilities.

Typically, research funding surges during outbreaks to address pressing gaps in knowledge about disease epidemiology and pathophysiology, and to accelerate development of diagnostics, treatments, vaccines, and vector control strategies. For example, the chikungunya epidemic in Réunion (2005-2006) spurred targeted research projects and fostered collaborative networks to tackle both fundamental and applied questions [19,20]. A decade later, dedicated international funding in response to the Zika pandemic generated major advances in flavivirus biology, illuminated the economic and societal burden of these diseases, and propelled development of rapid diagnostics, vaccine candidates, and novel vector control tools such as the Sterile Insect Technique.

However, as reported in the literature [21], large-scale, outbreak-driven funding often comes at the expense of ongoing research initiatives. Once the immediate threat subsides and public, media, and political attention wanes, funding is frequently reallocated, resulting in promising projects being postponed or abandoned until the next epidemic surge. For *Aedes*-borne arboviral diseases, prevention investments—including research—remain modest, accounting for less than 10% of the economic damages and losses caused by these diseases [22].

## Society

From intensity level 2 onward, an outbreak/epidemic would affect society as a whole. First, social movements challenging public health policy may develop, particularly groups opposing vector control activities, which could influence the intensity of transmission. As highlighted in several questionnaires, individual and collective opposition has already been observed, though rarely, in the DROMs and mainland France (Supplement B, Table B.1, rows D5-1 to D5-4). However, vector control activities are generally received positively by the population, and the emergence or development of groups calling for nuisance or vector control activities can also be envisaged. We also anticipate the development of associations of patients and people affected by the diseases. Finally, in the event that health authorities issue recommendations for vaccination in the longer run, our research team expects that social movements opposing vaccines may emerge, driven by misinformation.

A central question is whether an arboviral epidemic would exacerbate social inequalities. Given the limited research on the nexus between arboviruses and inequality, it is tempting to draw parallels with the coronavirus disease (COVID) pandemic (which began in 2019).^23^ However, such comparisons should be made with caution.

For arboviroses, the few studies available suggest that arboviral epidemics exacerbate territorial, socioeconomic, and health inequalities [23]. In the event of an outbreak/epidemic, the distribution of environmental or climatic characteristics in a given area (department or smaller area) may have an effect on the incidence, prevalence, and even mortality within the population. For instance, suburban areas favor the presence of breeding sites, because of the presence of gardens, flower pots, and gutters.

The literature highlights the interplay between environmental and socioeconomic factors (e.g. income poverty, poor housing conditions, and migration status) in shaping arboviral transmission [24,25]. More favorable socioeconomic and behavioral factors, including the utilization of air-conditioning, proper waste disposal, and the use of mosquito nets and fans, are associated with a reduction of transmission [24]. On a related matter, questionnaires from RHAs in the DROMs indicate that individuals experiencing social isolation are particularly vulnerable (Supplement B, Table B.1, row D5-5), highlighting the multidimensional nature of vulnerability in an outbreak/epidemic context.

## Information and misinformation

The occurrence of an epidemic/outbreak would likely be widely relayed not only by traditional media, but also by social media that enable faster and broader dissemination of information [26]. The way information is framed and disseminated by the media influences public opinion and perception of risk, shaping the behaviors of citizens, patients, and medical staff related to prevention and care [27]. The dissemination of information can either mitigate or amplify an outbreak/epidemic and its effects, according to stakeholders (e.g. Supplement B, Table B.1, row D3-4) and our own assessment. These effects on information are expected to emerge from transmission intensity level 2 onward.

**Figure 3:**
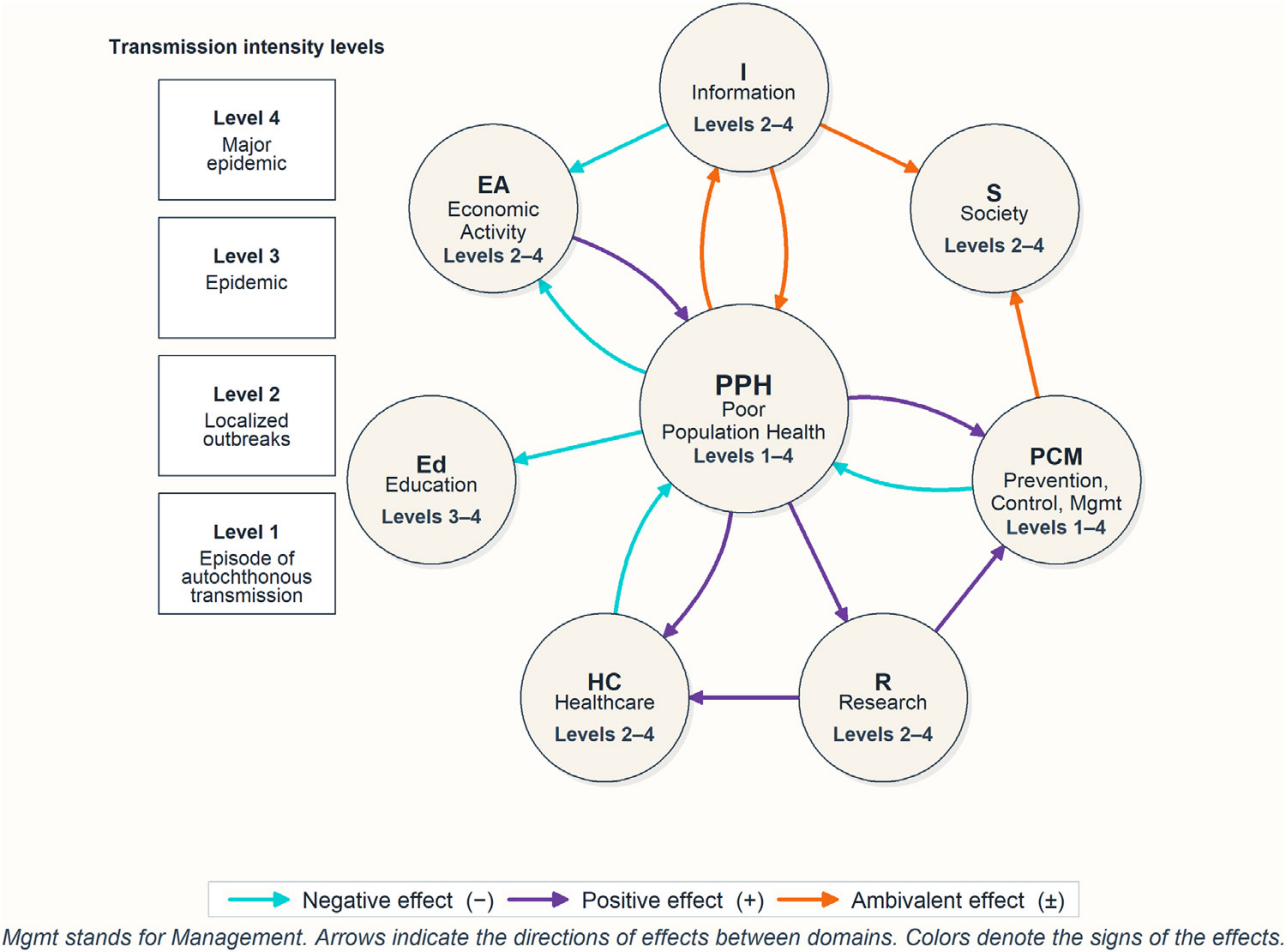
Inter-domain relationships.

The previous sections have examined the multifaceted impacts of an outbreak/epidemic on population health, the healthcare system, economic activity, education, research, and society at large. We have highlighted the role of information and misinformation in shaping the course and perception of the crisis. Figure 3 illustrates the relationships between domains in which effects could be observed. Table 2 is presented alongside the figure to provide a description of the relationships. Several feedback loops are identified: between prevention, control, and management and population health, between population health and healthcare, between population health and economic activity, and between population health and information.

**Table 2:** Description of inter-domain relationships.

Effect	Interpretation	Sign of the effect	Inter-domain loop
PPH –> PCM	Cases drive the implementation of prevention, control, and management strategies, until saturation of vector control resources.	Positive until saturation of vector control resources	Loop
PCM –> PPH	Efforts in prevention, control, and management reduce the number of cases, until this is no longer a public health emergency.	Negative until this is no longer a public health emergency	
PCM –> S	Control interventions try to increase social mobilization. However, there may be opposition movements to vector control and management.	Ambivalent	
PPH –> HC	Poorer population health leads to increased healthcare demand, until saturation of the healthcare system.	Positive until healthcare saturation	Loop
HC –> PPH	A better functioning healthcare system improves population health (i.e. decreases poor health). However, a saturated healthcare system is less effective in protecting population health.	Negative before healthcare saturation	
PPH –> EA	Poorer health leads to an increase in work absenteeism, which translates into a decrease in economic activity.	Negative	Loop^a^
EA –> PPH	Tourism (human mobility) contributes to the spread of an outbreak/epidemic.	Positive	
PPH –> Ed	The deterioration of population health goes hand in hand with absences among pupils and teachers.	Negative	
PPH –> R	The epidemic results in intensified efforts in research.	Positive	
R –> HC	Research translates into new treatments and diagnostic tools.	Positive	
R –> PCM	Research contributes to the improvement of vector monitoring and control strategies.	Positive	
PPH –> I	An outbreak/epidemic drives the generation and dissemination of health-related information. The information may be either accurate or inaccurate.	Ambivalent	Loop
I –> PPH	High-quality information enhances the adoption of preventive behaviors. In contrast, misinformation fuels opposition to prevention.	Ambivalent	
I –> EA	Information related to an outbreak/epidemic has a negative effect on tourism.	Negative	
I –> S	Information affects society by increasing awareness of health risks (social movements, collective actions).	Ambivalent	

a Each effect flows in one direction without returning to influence the initial cause.

## Policy recommendations

To prevent or mitigate the detrimental effects of an outbreak/epidemic in multiple domains, several intersectoral policy recommendations are outlined in Supplement D. While they reflect a proactive and/or preventive approach (rather than a reactive response to a crisis), these recommendations emphasize the necessity of collaborations between stakeholders from different sectors.

## Conclusion and discussion

Given arboviral risks in temperate zones, fueled by climate change and global mobility, this paper explores the potential multisectoral effects of arboviral outbreaks and epidemics, in mainland France. We identify potential effects in several domains including public health policy, population health, healthcare, economic activity, education, research, society, and the media.

Of note, we observe that absenteeism may serve as a channel through which an epidemic may produce effects across various domains (absenteeism among professionals in RHAs and mosquito control operators, healthcare workers, teachers, and students) with cross-cutting impacts.

We identify several critical vulnerabilities, including the potential saturation of vector control resources and the risk of overburdening healthcare services. The risk of saturation among RHAs and vector control operators (which may escalate further if the operators themselves were to be affected by the viruses) poses a risk of uncontrolled transmission, and calls for greater financial and human resources for prevention, management, and control. This also underscores the need for further reflection on the evolution of vector control, such as alternative approaches to chemical treatment and a greater engagement of the population. To prevent or mitigate healthcare system saturation, the experience of the DROMs highlights the importance of anticipating the reorganization of care (e.g. a dedicated emergency pathway for arboviral patients, to prevent saturation of the emergency department—Supplement B, Table B.1, rows D2-2 to D2-4).

Other major vulnerabilities are apparent, such as economic losses (especially in the tourism industry) and social disruption (in case of social movements challenging public policies). We emphasize gaps in preparedness, mainly driven by the low awareness of arboviral threats among the population and healthcare professionals. Raising healthcare professional awareness of risk factors and warning signs associated with severe cases remains a critical issue.

Epidemic events may also affect the education domain, challenging the ability of authorities to anticipate and manage teacher shortages, costs linked to mosquito control and school closures, and the adaptation of educational programs (Supplement B, Table B.1, row D4-3).

The ability of public authorities to manage information is a key issue. On the one hand, accurate and well-targeted communication can encourage community engagement and mobilization, potentially reducing transmission risk. On the other hand, poorly managed information may increase fear and panic, increasing detrimental effects. For public authorities, analyzing social media activity related to the outbreak/epidemic constitutes a valuable tool for assessing the dynamics of public opinion. Such analysis enables a real-time understanding of citizens’ concerns and perceptions, and tracking the spread of misinformation, thereby informing more responsive and targeted communication strategies [28].

Our analysis also reveals that arboviral outbreaks/epidemics may generate both balancing and reinforcing feedback loops (Figure 3 and Table 2). Policies must prioritize leverage points: strengthening virtuous loops (e.g. loops between disease cases and vector management, between cases and a well-functioning healthcare system, and between cases and accurate health information) while mitigating harmful ones (e.g. between cases and vector control saturation, between cases and healthcare overload, and between cases and misinformation). A coordinated, cross-sectoral approach is essential to break the riskiest dynamics. Our expert team further concludes that the most effective means to minimize both the risk of an outbreak/epidemic and the scale of its multisectoral effects would be to strengthen the development and implementation of integrated vector management.

Our analyses suffer from a number of limitations. First, because of a lack of data and academic publications (for comparable areas), our analysis of the potential effects of an outbreak/epidemic is predominantly prospective and inherently subject to uncertainty. Our approach is mainly qualitative and we are unable to provide a quantitative assessment of the effects we identify. Second, predicting the health burden (in disability-adjusted life years--DALYs) of an outbreak/epidemic in mainland France cannot rely on estimates from the DROMs or other countries. No DALY estimates for dengue, chikungunya, and Zika infections are available for the DROMs, and differences in population immunity further limit comparability. Available estimates from other countries (e.g. [29] for dengue) are also unsuitable, because of differences in immunity, comorbidities, surveillance, response, and healthcare system. In the perspective of a future outbreak in Europe, careful data collection should be planned in all affected domains we identify and supported by stable research programs.

The way our multidisciplinary expert team works highlights the importance of the dialogue between research fields studying outbreaks/epidemics and their effects. The implementation of an interministerial and cross-sectoral crisis management plan to anticipate and reduce the effects of outbreaks/epidemics in multiple domains is a key recommendation from our expert team.

## Declaration of competing interests

We declare no competing interests.

## Appendix - Supplementary Material

Image, application 1

## Footnotes

Supplementary material associated with this article can be found, in the online version, at doi:10.1016/j.ijregi.2026.100922.

## Footnotes

1 See: European Centre for Disease Prevention and Control, https://www.ecdc.europa.eu/en/all-topics-z/dengue/surveillance-and-disease-data/autochthonous-transmission-dengue-virus-eueea; (accessed 10 May 2025).

2 A department (or “département” in French) is an administrative division in France. France is divided into 101 départements, 94 of which are located in mainland France.

3 See: https://www.santepubliquefrance.fr/maladies-et-traumatismes/maladies-a-transmission-vectorielle/chikungunya/articles/donnees-en-france-metropolitaine/chikungunya-dengue-et-zika-donnees-de-la-surveillance-renforcee-en-france-metropolitaine-en-2022; (accessed 10 May 2025).

4 See: https://www.santepubliquefrance.fr/maladies-et-traumatismes/maladies-a-transmission-vectorielle/chikungunya/articles/donnees-en-france-metropolitaine/chikungunya-dengue-et-zika-donnees-de-la-surveillance-renforcee-en-france-metropolitaine-en-2023; (accessed 10 May 2025).

5 See: https://www.santepubliquefrance.fr/maladies-et-traumatismes/maladies-a-transmission-vectorielle/chikungunya/articles/donnees-en-france-metropolitaine/chikungunya-dengue-et-zika-donnees-de-la-surveillance-renforcee-en-france-hexagonale-2024; (accessed 10 May 2025).

6 See: European Centre for Disease Prevention and Control, https://www.ecdc.europa.eu/en/infectious-disease-topics/chikungunya-virus-disease/surveillance-and-updates/local-transmission-previous-years; (accessed 24 February 2026).

7 See: https://www.santepubliquefrance.fr/maladies-et-traumatismes/maladies-a-transmission-vectorielle/chikungunya/documents/bulletin-national/chikungunya-dengue-zika-et-west-nile-en-france-hexagonale.-bulletin-de-la-surveillance-renforcee-du-26-novembre-2025; (accessed 13 February 2026).

8 See: European Centre for Disease Prevention and Control, https://www.ecdc.europa.eu/sites/default/files/documents/ZIKV_AER_2022_Report.pdf; (accessed 24 July 2025).

9 We estimated that the likelihood of a level 3 epidemic (as defined in Supplement C), occurring within the next five years, is between 6 and 7 on a 0-9 scale. The uncertainty is equal to 2, which is considered average.

10 There are five DROMs: Guadeloupe, Martinique, French Guiana, Réunion, and Mayotte.

11 Guadeloupe, Martinique, Mayotte, Réunion.

12 French Guiana.

13 See: https://www.santepubliquefrance.fr/maladies-et-traumatismes/diabete/documents/article/prevalence-du-diabete-connu-a-la-reunion-prises-en-charge-et-caracteristiques-des-personnes-atteintes-d-un-diabete-exploitation-des-donnees-du-b; (accessed 13 February 2026).

14 See: https://www.santepubliquefrance.fr/presse/2023/sante-perinatale-en-2021-des-indicateurs-ultra-marins-globalement-plus-defavorables-qu-en-hexagone; (accessed 13 February 2026).

15 See: https://www.santepubliquefrance.fr/regions/ocean-indien/documents/article/2012/la-drepanocytose-dans-les-departements-francais-d-outre-mer-antilles-guyane-la-reunion-mayotte-.-donnees-descriptives-et-organisation-de-la-pri; (accessed 13 February 2026).

16 “Déclaration obligatoire.”

17 “Santé publique France.”

18 “Plan ORSEC-LAV” (“Organisation de la réponse de sécurité civile - Lutte antivectorielle”).

19 See: https://www.coreb.infectiologie.com/UserFiles/File/actus/2023-symptomes-et-evolution-de-la-dengue-vars20230923.pdf; (accessed 27 April 2026).

20 A stakeholder compares the potential effect of an epidemic on economic activity, in mainland France, with that of an influenza or COVID epidemic (Supplement B, Table B.1, row D3-1).

21 The literature provides some evidence on the effects on economic activity in tropical regions, highlighting in particular market resilience in Latin American and Caribbean countries after a Zika virus outbreak [4] and a GDP loss of 0.02% in 2013 in Brazil because of a dengue epidemic [5]. However, these results are not readily applicable to the context of mainland France.

22 For dengue in Singapore (2010-2020), productivity losses may exceed 75% of the total epidemic cost [6].

23 During the chikungunya epidemic in Réunion, the press made direct reference to the COVID crisis. See: https://www.lemonde.fr/planete/article/2025/04/18/l-epidemie-de-chikungunya-ebranle-la-reunion-il-s-agit-d-un-mini-covid-a-l-echelon-local_6597448_3244.html; (accessed 26 May 2025).
