# Supplementary material for: From bites to ripple effects: Unraveling the health, economic, and social effects of arboviral epidemics in Mainland France
Source: IJID Reg. 2026 May 25;20:100922. doi: 10.1016/j.ijregi.2026.100922 (PMC13315096; doi:10.1016/j.ijregi.2026.100922)
Supplement: Supplementary file 1 [file mmc1.pdf]

FROM BITES TO RIPPLE EFFECTS: UNRAVELING THE HEALTH, ECONOMIC, AND SOCIAL EFFECTS OF ARBOVIRAL EPIDEMICS IN MAINLAND FRANCE

SUPPLEMENTARY MATERIAL

May 1, 2026

Contents

1 SUPPL. A: MATERIAL AND METHODS **2**

1.1 Systematic literature review and complementary opportunistic search . . . 2

1.2 Qualitative data . . . 5

1.2.1 Questionnaire to regional mosquito control operators (mainland France) . . . 9

1.2.2 Questionnaire to Regional Health Agencies (DROMs) . . . 11

1.2.3 Questionnaire to Regional Health Agencies (mainland France) . . . 13

1.2.4 Questionnaire to clinicians in hospitals (DROMs) . . . 16

1.2.5 Questionnnaire to academic hospital managers (mainland France) . . . 18

1.2.6 Questionnnaire to the French Blood Agency . . . 19

1.2.7 Questionnnaire to Chambers of Commerce and Industry (DROMs) . . . 20

1.2.8 Questionnnaire to Chambers of Commerce and Industry (mainland France) . . . 21

2 SUPPL. B: VERBATIM QUOTATIONS DRAWN FROM THE QUESTIONNAIRE RESPONSES **22**

3 SUPPL. C: EFFECTS BY TRANSMISSION INTENSITY LEVELS **26**

4 SUPPL. D: INTERSECTORAL POLICY RECOMMENDATIONS **27**

1 SUPPL. A: MATERIAL AND METHODS

There has been no significant epidemic in Europe (beyond level 2 as defined in Suppl. C), and no study has assessed the effects of an outbreak or epidemic due to an arbovirus transmitted by *Ae. albopictus*, in Europe or in a temperate country. Given the lack of literature on temperate regions, and because evidence from equatorial or tropical settings is not directly transferable to mainland France, we developped an original framework to anticipate the potential effects of such an epidemic, grounded on triangulation, combining a systematic literature review, an opportunistic search, the consultation of stakeholders, and our expert input (derived from discussions among us and our own professional and research experience).

1.1 Systematic literature review and complementary opportunistic search

Our objective was to identify the full range of potential socioeconomic impacts, by answering the following question:

> *What would be the socioeconomic impacts of an outbreak or epidemic due to an arbovirus transmitted by Ae. albopictus in mainland France?*

To identify all research related to the socioeconomic impacts of arboviroses, we carried out a systematic literature review[1] using the PICO (Population, Intervention, Comparators, Outcomes) method.[2] The population was defined as "patients infected by an arbovirus transmitted by *Ae. albopictus* (i.e. dengue, Zika, and chikungunya viruses)" and outcomes as "socioeconomic impacts."

In November 2023, bibliographic searches were carried out on Scopus, PubMed, and Cairn, with no restriction on the year of publication. Our search strategy, results, and search date, for each database, are presented in Table A.1. The same search string in English was used for both PubMed and Scopus, wheras a very similar search string in French was applied to Cairn. The literature search included all relevant publications available up to the search dates (November 13 or 14, 2023).

Database	Search string	Results	Search date	Comments
PubMed	((social OR economic OR socioeconomic) AND (burden OR effect* OR impact)) AND ((arbovirus* AND aedes) OR dengue OR chikungunya OR zika)	1862	November 13, 2023	Keywords in the title only
Scopus	((social OR economic OR socioeconomic) AND (burden OR effect* OR impact)) AND ((arbovirus* AND aedes) OR dengue OR chikungunya OR zika)	87	November 14, 2023	Keywords in the title only
Cairn	((soc* OU economi* OU socioeconomi*) ET (fardeau OU effet OU impact)) ET ((arbovir* ET aedes) OU dengue OU chikungunya OU zika)	13	November 14, 2023	Keywords in French. Keywords related to economic and impact were searched for in the title, whereas keywords related to arbovirus were searched for in the abstract

Table A.1: Literature search strategy and results

The selection of references for inclusion in the bibliographic review was carried out on the CADIMA website (CADIMA®), with a first stage involving screening of titles and abstracts, followed by a second stage involving full-text review of the articles. The selection took into account the PICO criteria presented above, as well as the additional eligibility criteria presented in Table A.2.

Level	Domain	Inclusion criteria	Exclusion criteria
Title / Abstract / Full text	Language	French or English or Spanish	
Title / Abstract / Full text	Topic	Studies related to dengue, chikungunya or Zika (i.e., viruses transmitted by *Aedes albopictus*)	Studies only related to Yellow Fever or to other mosquitoes (e.g., *Culex*, *Anopheles*)
Title / Abstract / Full text	Outcome / Design	Studies related to the impact or the burden of an outbreak or epidemic	Descriptive epidemiological studies if they only report the number of cases; studies evaluating the effect of risk factors on epidemics; studies evaluating the cost of prevention measures
Title / Abstract / Full text	Setting	Studies related to a geographical area similar to mainland France; studies conducted in low-income countries reporting novel impacts not previously identified by the expert group	Studies conducted in low-income regions reporting no novel impacts

Table A.2: Additional eligibility criteria

We noted, during our systematic literature review, that the number of academic/peer-reviewed studies on socioeconomic impacts was limited. For this reason, and because our aim was to identify impacts rather than quantify them, we retained all sources from the three databases that met our criteria, regardless of their methodological quality.

At each selection stage, each reference was reviewed by two readers independently, and any discrepancies in selection were resolved through the interface available on CADIMA.

Data extraction was conducted in an exploratory manner. For each included study, we extracted the following information: country, virus, study design (e.g., observational study, retrospective data analysis), and outcome. The synthesis followed a narrative approach. Reported impacts were grouped thematically, and particular attention was paid to whether similar impacts were described across multiple studies.

In addition, we conducted an opportunistic search to identify information not captured in the systematic search, using three different strategies. First, we included relevant academic articles and institutional reports known to the research team that were missed in the systematic search. Second, we conducted Google Scholar searches using a search string with the following structure: “socioeconomic impact” or a specific type of impact (e.g., “tourism”) followed by a disease (dengue, chikungunya, or Zika). Third, we searched for newspaper articles using Google. This opportunistic search yielded 58 academic articles, as well as some institutional reports and newspaper articles.

The following flow chart illustrates the process followed in conducting the literature review, outlining the key stages of selection. The 58 additional academic articles are shown in the flow chart.

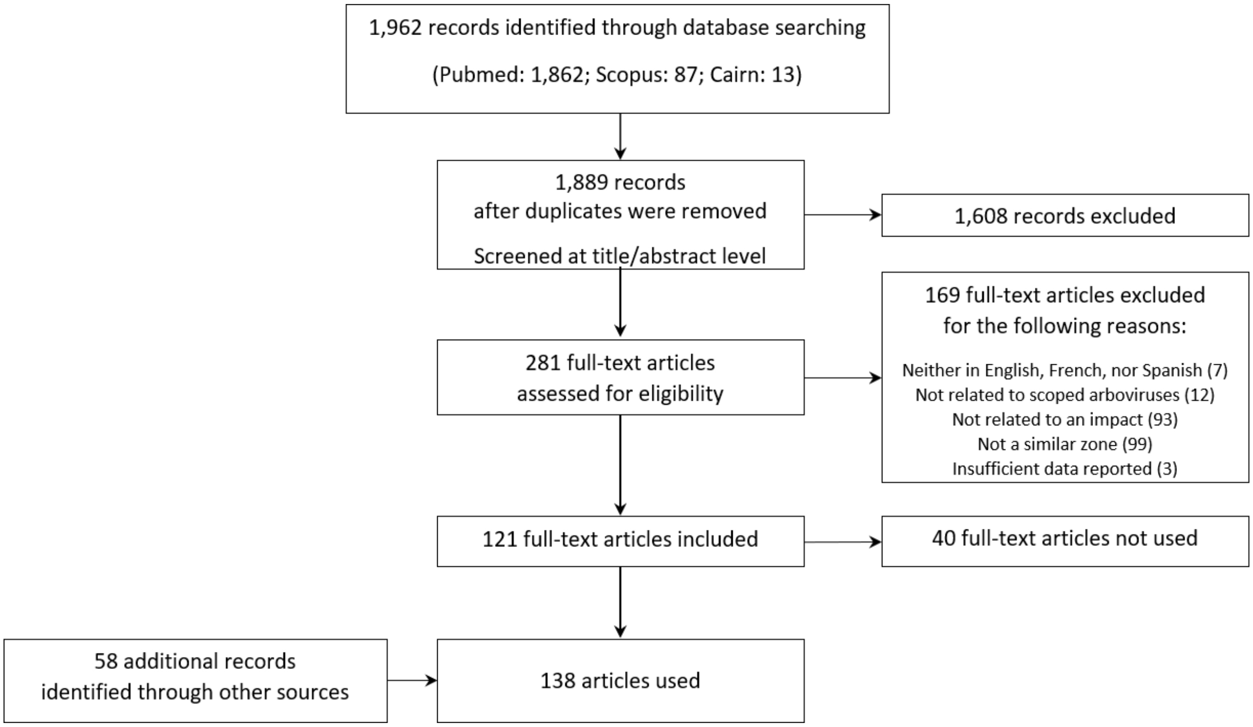

Figure A.1: Flow chart of study selection

1.2 Qualitative data

In parallel, we gathered qualitative data that extend beyond what is available in the scientific literature. We first identified stakeholders, i.e. individuals or organizations that had previously experienced an arboviral epidemic in the DROMs, or that were likely to be directly affected by an outbreak or epidemic in mainland France: regional mosquito control operators, Regional Health Agencies (RHAs), clinicians in hospitals, academic hospital managers, the French Blood Agency, and Chambers of Commerce and Industry (CCIs). We sent a questionnaire to all RHAs, except one in the DROMs. One RHA from the DROMs did not respond. In total we received responses from 16 RHAs (three for the DROMs and 13 for mainland France). For clinicians, we made sure to include respondents who had faced an epidemic in the Caribbean and in La Réunion. We surveyed CCIs to gather insights on the (potential) economic effects of outbreaks/epidemics on local businesses and commercial activities.

The questionnaires, containing open-ended questions that we designed to be relevant to each stakeholder's field of activity, are reported in Sections 1.2.1 to 1.2.8.

Stakeholders were first contacted by email. Two reminder emails were sent to non-respondents. An additional reminder email was sent to clinicians who had not responded. If a RHA did not respond after the second reminder, it was contacted by phone. The timeline for invitations and follow-ups is given in Table A.3.

Stakeholders	Date of first contact (email)	Responses received / Questionnaires sent	Date of first reminder* (email)	Responses received / Questionnaires sent	Date of second reminder* (email)	Third reminder*	Responses received / Questionnaires sent
Regional mosquito control operators	November 30, 2023	6 / 9	December 15, 2023	8 / 9	January 12, 2024	-	8 / 9
Regional Health Agencies (RHAs), DROMs	November 30, 2023	1 / 4	December 15, 2023	2 / 4	January 12, 2024	(Phone)	3 / 4
Regional Health Agencies (RHAs), mainland France	November 30, 2023	5 / 13	December 15, 2023	11 / 13	January 12, 2024	(Phone)	13 / 13
Clinicians in hospitals, DROMs	January 15, 2024	2 / 6	February 1, 2024	2 / 6	February 19, 2024	(Email) March 5, 2024	4 / 6
Academic hospitals managers, mainland France	January 26, 2024	1 / 8	February 12, 2024	5 / 8	March 5, 2024	-	6 / 8
French Blood Agency	January 26, 2024	1 / 1	-	-	-	-	1 / 1
Chambers of Commerce and Industry (CCIs)	January 26, 2024	0 / 6	February 12, 2024	1 / 6	March 5, 2024	-	2 / 6

Table A.3: Timeline of questionnaires responses

Notes: * Only non-respondents were recontacted (by email or phone).

Table A.4 presents the list of stakeholders, the number of respondents and non-respondents, and the topics covered in the questionnaires.

Stakeholders (French)	Mainland France or DROMs	Topics covered in the questionnaire	Number of respondents	Number of non-respondents
Regional mosquito control operators ("Opérateurs de démoustication" – OpD)	Mainland France	Organizational context; operational difficulties and constraints; public reactions and reluctance; vulnerabilities; operational capacity; saturation and impacts	8	1
Regional Health Agencies – RHAs ("Agences régionales de santé" – ARS)	DROMs	Public reactions and reluctance; impact on healthcare services, economic activity and other domains; factors influencing impact; monitoring and crisis management; vulnerabilities	3	1
	Mainland France	Organizational context; prevention and vector control strategy; operational capacity and constraints; vector control operations; impact on healthcare services, economic activity and other domains; monitoring and crisis management	13	0
Clinicians in hospitals	DROMs	Impact on healthcare services; saturation and consequences; factors influencing impact; impact on healthcare; vulnerabilities; nosocomial transmission and prevention; long-term consequences and inequalities	4	2
Academic hospitals managers ("Direction de Centres hospitaliers universitaires" – CHU)	Mainland France	Preparedness and organizational adaptations; nosocomial transmission and prevention; operational constraints	6	2
French Blood Agency ("Établissement français du sang" – EFS)	-	Tests; operational strategies; costs; impact of an epidemic on the availability of blood products	1	1
Chambers of Commerce and Industry ("Chambres de commerce et d'industrie" – CCI)	DROMs	Risk perception and preparedness; economic impact and vulnerabilities; organizational adaptations; economic opportunities	1	3
	Mainland France		1	1

Table A.4: Questionnaires sent and completed by stakeholders

For the analysis, we began by compiling all responses to the questionnaires into a single table. Given the limited number of respondents, we did not use software for formal qualitative analysis. Several researchers from our expert team independently read and analyzed the responses, combining deductive categories (prevention, control, and management / health / healthcare / economic activity / education / research / society / information and misinformation) and inductive themes emerging from the data. We then discussed collectively to interpret the findings; any coding discrepancies were resolved by consensus. The qualitative data we collected allowed us to gain insights into the potential effects of an outbreak/epidemic.

1.2.1 Questionnaire to regional mosquito control operators (mainland France)

Questionnaire pour les opérateurs de LAV

Notre interrogation porte sur les éléments qui peuvent limiter votre action dans la lutte antivectorielle (LAV) aujourd'hui – enquête entomologique et traitement éventuel – et sur les impacts potentiels d'une augmentation de la transmission autochtone d'arboviroses par *Aedes albopictus* dans l'Hexagone.

Vous pouvez également nous transmettre tout document qui apporterait ou compléterait les réponses aux questions posées.

1. Contexte

Par rapport au schéma ci-dessous, pouvez-vous indiquer s'il correspond à votre activité et si vous interagissez avec d'autres acteurs non mentionnés pour l'activité qui relève de la LAV ?

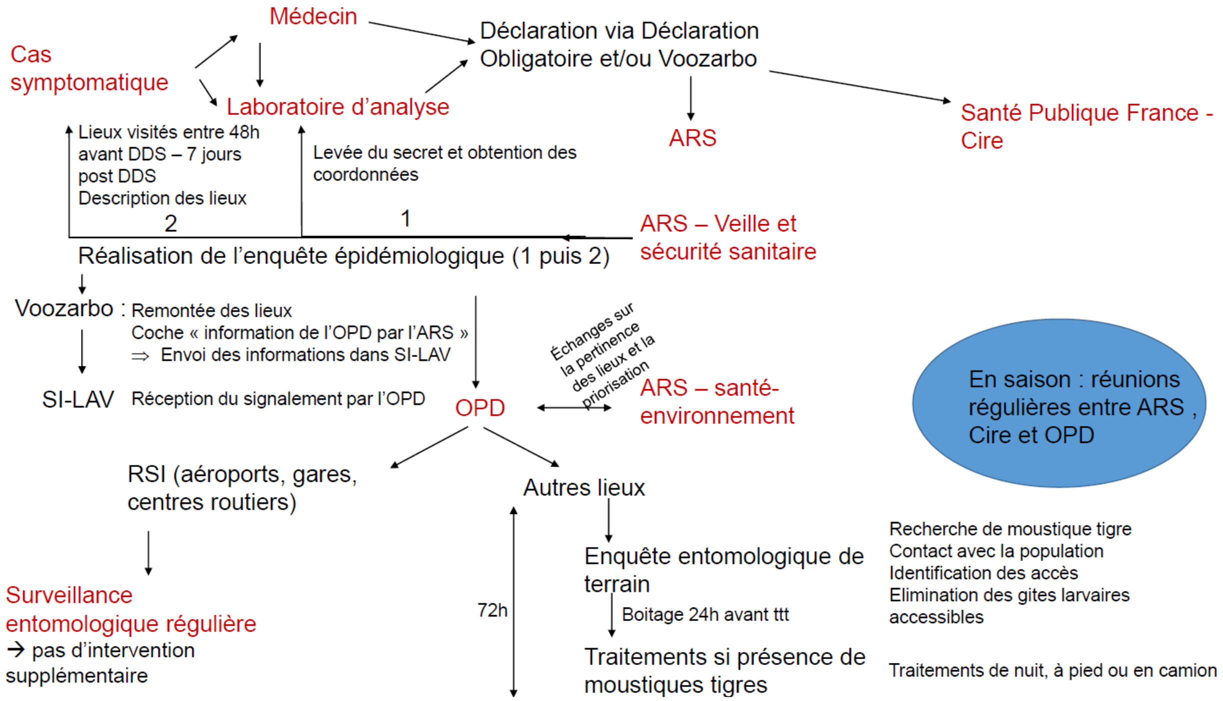

2. Activité actuelle

2.1. Vous heurtez-vous à des difficultés dans la mise en œuvre de vos missions de lutte antivectorielle ? Si oui, lesquelles ?

2.2. Avez-vous accès et pouvez-vous traiter l'ensemble des gîtes larvaires et lieux de repos prévus dans votre protocole de traitement ?

2.3. Quelles sont les réactions (positives, neutres ou négatives) dans la population lors de vos interventions ? Observez-vous des réticences ?

2.4. Si vous vous heurtez à des réticences,

- Sur quelles interventions portent-elles (suppression des gîtes larvaires, traitement adulticide, piégeage) ?
- Quels motifs sont exprimés ?
- D'où viennent les réticences (par ex. habitants, acteurs économiques, apiculteurs, acteurs politiques, militants associatifs) ?

2.5. Ces réticences ont-elles un impact sur votre organisation (besoin de formation complémentaire par exemple) ou sur la façon de mener votre activité (délai ou impossibilité d'effectuer des traitements) ?
2.6. Avez-vous des informations sur la part des traitements qui ne sont pas complètement réalisés, du fait de réticences ou pour d'autres motifs ?
2.7. Y a-t-il d'autres motifs qui peuvent gêner votre activité ?
2.8. Identifiez-vous des environnements / habitants / zones particulièrement vulnérables au risque de transmission autochtone ?

3. Perspective d'augmentation de l'activité
3.1. Vos moyens (humains, matériels) sont-ils mutualisés sur plusieurs territoires ? Si oui, à quelle échelle (entre départements, entre régions, sur tout le territoire national) ?
3.2. Jusqu'à quel niveau d'activité êtes-vous engagés par le(s) marché(s) public(s) qui vous est (sont) attribué(s), selon les zones géographiques concernées ?
3.3. Jusqu'à quel niveau d'activité pourriez-vous répondre à une demande supplémentaire et/ou exceptionnelle ? Le cas échéant, quels changements pourriez-vous envisager pour répondre à cette demande supplémentaire et dans quel délai (par exemple, réallocation des moyens internes, recrutements, formation, mutualisation des moyens avec d'autres opérateurs, sous-traitance) ? Quelles seraient les conditions nécessaires à cette adaptation (moyens supplémentaires, délais par exemple) ?
3.4. Quelles seraient d'après vous les conséquences de cette réorganisation (pour vous ou pour votre activité) ?
3.5. À partir de quel niveau (en nombre de cas importés ou autochtones, ou de sites à traiter ou de foyers) pensez-vous atteindre vos limites de capacité à intervenir y compris après adaptation ? Quels seraient les freins principaux à une augmentation de votre activité (financement, moyens matériels, moyens humains, ...) ?
3.6. Quels seraient selon vous les impacts d'une saturation de vos moyens ?

4. Commentaires libres
Avez-vous des commentaires à partager sur la mise en œuvre de la LAV, dans la perspective d'une augmentation du nombre de cas d'arboviroses transmises par *Aedes albopictus* dans l'Hexagone ?

1.2.2 Questionnaire to Regional Health Agencies (DROMs)

Questionnaire pour les ARS des DROM

Nos interrogations portent sur la gestion et les impacts des arboviroses transmises par *Aedes albopictus* (dengue, chikungunya, Zika). N'hésitez pas à moduler vos réponses selon le virus si nécessaire.

Vous pouvez également nous transmettre tout document qui apporterait ou compléterait les réponses aux questions posées.

1. Question relative à l'activité de lutte antivectorielle
Faites-vous face à des réactions de la population lors de la mise en œuvre des traitements de LAV ? Si oui, de quel type, sous quelle forme ?

2. Questions relatives à l'impact des arboviroses sur l'offre et l'organisation des soins et sur les autres activités
2.1. Quels domaines de l'offre de soins sont les plus touchés par une épidémie de dengue / Zika / chikungunya : médecine générale, services d'urgence, hospitalisation, pharmacies, laboratoires, infirmiers, autres (préciser) ?
2.2. Parmi les acteurs les plus touchés, à partir de quel niveau de transmission (ou d'activité, par exemple à partir de combien de passages aux urgences) pensez-vous que les maladies transmises par les moustiques *Aedes* (dengue, Zika, chikungunya) auraient un impact significatif (de nature à modifier l'activité habituelle) :

- Sur l'offre de soins (soins curatifs ambulatoires et hospitaliers, diagnostic biologique) ? En particulier, à partir de combien de cas ou de quel niveau de transmission, pensez-vous que l'offre de soins est / serait saturée (selon le type de ressources et le contexte – territoire, offre de soins initiale) ?
- Sur l'organisation des soins (nécessité de modifier l'offre de soins) ?
- En termes économiques :
  - sur les activités des entreprises (absentéisme des employés) ?
  - sur les individus (incapacité à travailler et/ou aide des malades, garde d'enfants) ?
  - sur d'autres acteurs : école par exemple (absence des enseignants/fermeture ponctuelle, autres) ?
- Autres ?

2.3. Quels sont les facteurs qui peuvent modifier ce niveau de saturation à la baisse ou à la hausse (par ex. offre de soins initiale, information de la population, sensibilisation des professionnels de santé, gestion d'une épidémie d'arbovirose concomitante à une autre épidémie...) ?
2.4. En phase épidémique,

- quels indicateurs sanitaires ou liés à l'offre de soins surveillez-vous (passages aux urgences, hospitalisations...) ?
- quelle organisation mettez-vous en place en cas de saturation de l'offre de soins ?

2.5. Identifiez-vous des populations particulièrement vulnérables face aux arboviroses ? Quels facteurs sont, selon vous, susceptibles de modifier les impacts sanitaires d'une épidémie d'arbovirose ?

3. Commentaires libres

Avez-vous des commentaires à partager sur les impacts des arboviroses, dans la perspective d'une augmentation du nombre de cas d'arboviroses transmises par *Aedes albopictus* dans l'Hexagone ?

1.2.3 Questionnaire to Regional Health Agencies (mainland France)

Questionnaire pour les ARS de l'Hexagone

Nos interrogations portent sur la gestion et les impacts des cas d'arboviroses transmises par *Aedes albopictus* (dengue, chikungunya, Zika) ainsi que les actions de LAV, du point de vue des ARS. N'hésitez pas à moduler vos réponses selon le virus si nécessaire.

Vous pouvez également nous transmettre tout document qui apporterait ou compléterait les réponses aux questions posées.

1. Contexte

Par rapport au schéma ci-dessous, pouvez-vous indiquer s'il correspond à votre activité et si vous interagissez avec d'autres acteurs non mentionnés pour l'activité qui relève de la gestion des cas et de la LAV ?

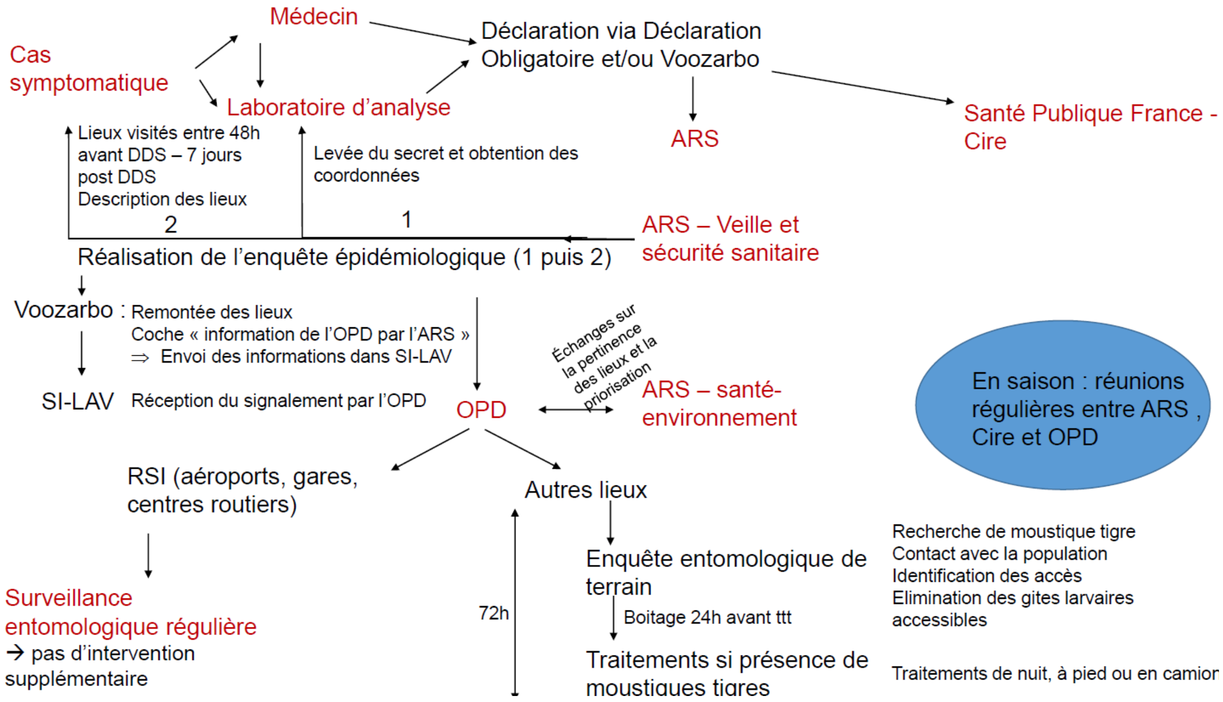

2. Questions générales relatives à la prévention et la lutte anti-vectorielle

2.1. De votre point de vue, y a-t-il des actions qui pourraient / devraient être mises en œuvre (et qui ne le sont pas) pour éviter ou limiter la transmission autochtone ? Si oui, lesquelles ? Certaines vous semblent-elles prioritaires ?

2.2. Quels sont les freins ou obstacles à la mise en œuvre des mesures de prévention et de lutte que vous citez ?

3. Questions relatives à l'activité d'investigation, de LAV et de prévention des cas d'arboviroses par les ARS (activité intra-ARS)

3.1. Vous heurtez-vous, dans votre organisation actuelle, à des difficultés dans la gestion (investigation et mise en œuvre des actions de prévention et de lutte) des cas importés ou des cas autochtones (par exemple, difficultés à joindre et interroger des personnes, réticences sur la communication autour des cas identifiés, difficultés à mettre en place les traitements, accès aux jardins des particuliers, etc.) ? Pouvez-vous décrire ces difficultés et quelles solutions vous pourriez envisager ?

3.2. Selon votre organisation actuelle, jusqu'à quel niveau d'activité (en nombre de cas importés, cas autochtones, nombre de foyers ou autre, par semaine) pouvez-vous assurer l'activité de suivi et de gestion des cas importés et autochtones ?

3.3. Quels sont les facteurs limitant votre capacité à gérer les cas (financiers, humains, champs de compétence, décisionnaires, autres) ?

3.4. Selon vous, quels seraient les impacts d'une saturation de vos moyens ? Est-il prévu le déblocage d'une enveloppe exceptionnelle ou une réorganisation d'urgence de vos moyens (par exemple, réallocation des moyens internes à l'ARS, recrutements ponctuels) ? Cette réorganisation aurait-elle un impact sur d'autres activités de l'ARS ? Quel serait l'impact opérationnel de cette réorganisation ?

3.5. Comment s'organisent les relais de gestion inter-ARS ou vers la Préfecture ?

3.6. Y a-t-il d'autres facteurs qui peuvent modifier votre capacité à assurer votre activité liée aux arboviroses (par exemple une sollicitation de vos ressources pour une autre demande) ?

4. Questions relatives à l'activité de lutte antivectorielle (par les opérateurs de démoustication)

4.1. Combien coûtent la surveillance entomologique et la LAV dans le cadre du marché public ?

4.2. Quels sont/ont été vos critères de choix d'opérateurs de LAV dans l'attribution du marché public et combien d'interventions sont prévues dans le marché (au total, par semaine) en situation normale ?

4.3. Si plusieurs opérateurs ont été retenus dans le marché public, comment mobilisez-vous les différents opérateurs ?

4.4. Avez-vous la possibilité de solliciter un opérateur de démoustication au-delà du cadre défini dans le marché (pour une activité supplémentaire et exceptionnelle par exemple) ? Si oui, dans quelles conditions (par exemple, avenant au marché public, priorisation des interventions, mobilisation de ressources supplémentaires, rapidité du processus) ?

4.5. Quels seraient d'après vous les impacts d'une saturation des moyens de LAV ?

4.6. Faites-vous face à des réactions de la population lors de la mise en œuvre des traitements ? Si oui, de quel type, sous quelle forme ?

5. Questions relatives à l'impact des arboviroses sur l'offre et l'organisation des soins, et les autres activités

5.1. Quel est selon vous le niveau de sensibilisation et de connaissance des professionnels de santé au risque d'arboviroses dans votre région (si possible, détaillez par type de professionnels : ville/établissements, infirmières/médecins...) ?

5.2. À partir de quel niveau de transmission (ou d'activité, par exemple à partir de combien de passages aux urgences) pensez-vous que les maladies transmises par les moustiques *Aedes* (dengue, Zika, chikungunya) auraient un impact significatif (de nature à modifier l'activité habituelle) :

- Sur l'offre de soins (soins curatifs ambulatoires et hospitaliers, diagnostic biologique) ? En particulier, à partir de combien de cas ou de quel niveau de transmission, pensez-vous que l'offre de soins serait saturée (selon le type de ressources et le contexte – territoire, offre de soins initiale) ?
- En termes économiques :
  - sur les activités des entreprises (absentéisme des employés) ?
  - sur les individus (incapacité à travailler et/ou aide des malades, garde d'enfants) ?
  - sur d'autres acteurs : école par exemple (absence des enseignants/fermeture ponctuelle, autres) ?
- Autres ?

5.3. Quels indicateurs de LAV, sanitaires ou liés à l'offre de soins suivez-vous ? Pouvez-vous / pourriez-vous être amené à imposer des mesures, à limiter une activité ou l'accès à certains lieux au cours d'un épisode de transmission autochtone d'arboviroses transmises par *Aedes albopictus* ? Si oui, selon quels indicateurs et seuils d'alerte ?

6. Commentaires libres

Avez-vous des commentaires à partager sur les impacts des arboviroses, dans la perspective d'une augmentation du nombre de cas d'arboviroses transmises par *Aedes albopictus* dans l'Hexagone ?

1.2.4 Questionnaire to clinicians in hospitals (DROMs)

Questionnaire pour les cliniciens dans les hôpitaux des DROM

Les questions visent à recueillir votre expérience de la gestion d'épidémies de dengue, chikungunya et/ou Zika afin d'estimer les impacts potentiels d'une augmentation de la transmission autochtone d'arboviroses par *Aedes albopictus* dans l'Hexagone.
Vous pouvez également nous transmettre tout document qui apporterait ou compléterait les réponses aux questions posées.

1. Quels secteurs de l'offre de soins sont touchés par une épidémie de dengue / zika / chikungunya (médecine générale, urgences, hospitalisation, laboratoires, infirmiers. . .) ? De quelle façon ?

2. À partir de quel seuil une épidémie a un impact significatif sur la prise en charge (des personnes souffrant d'arbovirose et des personnes recourant aux soins pour d'autres motifs) ? Quels sont les facteurs limitant la prise en charge (par ex. disponibilité du personnel, nombre de lits, disponibilité du matériel...) ?

3. Quels sont les facteurs qui peuvent modifier ce seuil (par ex. information de la population, sensibilisation des professionnels de santé...) ?

4. Observez-vous une hausse du nombre d'arrêts de travail dans le personnel hospitalier lors d'une épidémie d'arbovirose ? Si oui, cette hausse perturbe-t-elle l'activité de votre établissement ?

5. Quels sont les évènements qui peuvent modifier de façon significative l'impact d'une arbovirose sur le système de soins et les soignants (par ex. concomitance d'une autre épidémie) ? Comment ?

6. Identifiez-vous des populations particulièrement vulnérables (d'un point de vue clinique) ?

7. Quels sont les évènements qui peuvent modifier de façon significative l'impact d'une arbovirose sur l'état de santé des populations (par ex. concomitance d'une autre épidémie) ? Comment ?

8. Avez-vous connaissance de transmission d'une arbovirose à du personnel hospitalier ou à des patients hospitalisés ou venus en consultation ? Existe-t-il des recommandations pour éviter une transmission à l'hôpital ? Quelles mesures de prévention de la transmission intra-hospitalière sont mises en œuvre ?

9. Quelles sont selon vous les populations les plus susceptibles de subir davantage les conséquences des arboviroses et pour quelles raisons (par exemple, du fait de difficultés d'accès aux soins, de manque d'information, de l'absence de soutien de proches, d'un reste à charge financier ou autre motif que vous pouvez préciser...) ?

10. Identifiez-vous des inégalités sociales liées aux effets à long terme du chikungunya (reste à charge / soins, aménagement du logement, aide pour les activités quotidiennes, capacité à travailler, revenu individuel...) ?

11. Y a-t-il des difficultés particulières à gérer une épidémie d'arbovirose concomitante à une autre épidémie ? Si oui lesquelles ?

12. Avez-vous d'autres commentaires à partager sur la prise en charge des patients lors d'une épidémie, dans la perspective d'une augmentation du nombre de cas d'arboviroses transmises par *Aedes albopictus* dans l'Hexagone ?

Nous vous remercions pour vos réponses, qui contribuent à alimenter la réflexion du groupe d'experts.

1.2.5 Questionnnaire to academic hospital managers (mainland France)

Questionnaire pour les CHU de l'Hexagone

Les questions visent à recueillir votre expérience dans la perspective de la gestion d'épidémies de dengue, chikungunya et/ou Zika afin d'estimer les impacts potentiels d'une augmentation de la transmission autochtone d'arboviroses par *Aedes albopictus* dans l'Hexagone.

Vous pouvez également nous transmettre tout document qui apporterait ou compléterait les réponses aux questions posées.

1. En cas d'épidémie d'arbovirose pouvant survenir pendant la période estivale et le début de l'automne (dengue, Zika, chikungunya), avez-vous anticipé une organisation spécifique des soins ? Quelles seraient pour vous les spécificités d'une telle épidémie en termes d'organisation de l'offre de soins, par rapport aux autres épidémies connues (grippe, bronchiolite...) ? Par exemple, profils des patients, épidémie d'une maladie inhabituelle en période estivale, personnel disponible, connaissance de la maladie par les professionnels, moyens diagnostiques, services concernés...

2. Avez-vous été sensibilisés au risque de transmission vectorielle des arboviroses qui surviendrait au sein de l'hôpital (transmission par piqûre de moustique des patients ou du personnel), en période épidémique ou non ? Si oui, comment ?

3. Mettez-vous en œuvre des mesures de prévention ? Si oui, lesquelles ? Existe-t-il un budget fléché pour financer ces mesures de prévention ? Le cas échéant, quels sont les obstacles à leur mise en œuvre ?

Nous vous remercions pour vos réponses, qui contribuent à alimenter la réflexion du groupe d'experts.

1.2.6 Questionnnaire to the French Blood Agency

Questionnaire pour l'Établissement français du sang

Les questions visent à recueillir votre expérience de la gestion d'épidémies de dengue, chikungunya et/ou Zika afin d'estimer les impacts potentiels d'une augmentation de la transmission autochtone d'arboviroses par *Aedes albopictus* dans l'Hexagone.

Vous pouvez également nous transmettre tout document qui apporterait ou compléterait les réponses aux questions posées.

1. Concernant les arboviroses transmises par *Aedes albopictus* en France hexagonale, quels tests de dépistage d'arboviroses pouvez-vous réaliser :

1.1. En cas d'épidémie ?

1.2. En cas de double épidémie d'arboviroses ?

2. Le dépistage serait-il individuel ou par pool ? (le cas échéant, détailler par arbovirose)

3. Quels seraient le coût des tests réalisés en cas d'épidémie (coût du test et coût logistique éventuel) ?

4. Quel serait l'impact d'une épidémie d'arbovirose sur la disponibilité des produits sanguins (suspension des collectes, retrait de dons) ? Pendant quelle durée ?

Nous vous remercions pour vos réponses, qui contribuent à alimenter la réflexion du groupe d'experts.

1.2.7 Questionnnaire to Chambers of Commerce and Industry (DROMs)

Questionnaire pour les CCI dans les DROM

Les questions visent à recueillir votre expérience de la gestion d'épidémies de dengue, chikungunya et/ou Zika afin d'estimer les impacts potentiels d'une augmentation de la transmission autochtone d'arboviroses par *Aedes albopictus* dans l'Hexagone.

Vous pouvez également nous transmettre tout document qui apporterait ou compléterait les réponses aux questions posées.

1. Le risque d'arboviroses (maladies transmises par les moustiques comme la dengue, le chikungunya ou le Zika) est-il un sujet de réflexion pour vous et les acteurs que vous accompagnez ?

2. D'après vous, quels secteurs d'activité ont été ou seront affectés par une épidémie d'arbovirose ? Pouvez-vous détailler pour chaque arbovirose (dengue, Zika, chikungunya) et chaque période? De quelle façon les secteurs ont-ils été ou seraient affectés ?

N'hésitez pas à nous communiquer toute donnée qui viendrait documenter votre expérience, en particulier des études d'impact sur l'activité économique réalisées lors d'épidémies précédentes (par exemple, évolution du chiffre d'affaires, faillites d'entreprises, emploi...).

3. Avez-vous observé ou anticipez-vous la mise en place de mesures en lien avec le risque d'arboviroses, à votre initiative ou celle des entreprises ?

4. Avez-vous observé ou anticipez-vous que le risque d'arbovirose puisse être une opportunité économique pour certaines entreprises (innovation, recherche et développement, commercialisation de tests diagnostiques ou de répulsifs, lutte antivectorielle, vaccins...) ?

Nous vous remercions pour vos réponses, qui contribuent à alimenter la réflexion du groupe d'experts.

1.2.8 Questionnnaire to Chambers of Commerce and Industry (mainland France)

Questionnaire pour les CCI en France hexagonale

Les questions visent à recueillir votre expérience dans la perspective de la gestion d'épidémies de dengue, chikungunya et/ou Zika afin d'estimer les impacts potentiels d'une augmentation de la transmission autochtone d'arboviroses par *Aedes albopictus* dans l'Hexagone.
Vous pouvez également nous transmettre tout document qui apporterait ou compléterait les réponses aux questions posées.

1. Le risque d'arboviroses (maladies transmises par les moustiques comme la dengue, le chikungunya ou le Zika) est-il un sujet de réflexion pour vous et les acteurs que vous accompagnez ?

2. D'après vous, quels secteurs d'activité seraient affectés par une épidémie d'arbovirose ? De quelle façon ?

3. Anticipez-vous la mise en place de mesures en lien avec le risque d'arboviroses, à votre initiative ou celle des entreprises ?

4. Pensez-vous que le risque d'arbovirose peut être une opportunité économique pour certaines entreprises (innovation, recherche et développement, commercialisation de tests diagnostiques ou de répulsifs, lutte antivectorielle, vaccins...) ?

Nous vous remercions pour vos réponses, qui contribuent à alimenter la réflexion du groupe d'experts.

2 SUPPL. B: VERBATIM QUOTATIONS DRAWN FROM THE QUESTIONNAIRE RESPONSES

The table below reports some of the stakeholders' responses to our questionnaires. The table is not exhaustive.

Row number	Stakeholder & original response in French	Stakeholder & response translated in English
	**PREVENTION, CONTROL, AND MANAGEMENT**	
D1-1	**Agence régionale de santé (ARS), France hexagonale.** Les moyens humains consacrés à la LAV [lutte anti-vectorielle] en ARS sont en forte hausse depuis ces dernières années. Les effectifs en ARS n'ayant pas été renforcés pour prendre en charge cette mission, voire réduits dans certains départements, les moyens humains constitueront un facteur limitant de la capacité à gérer les cas.	**Regional Health Agency (RHA), mainland France.** The human resources devoted to LAV [vector control] at the RHA have risen sharply in recent years. As RHA staff numbers have not been increased to take on this mission, and have even been reduced in some departments, human resources will be a limiting factor in the ability to manage cases.
D1-2	**Agence régionale de santé (ARS), France hexagonale.** En 2023, un à deux cas par jour pendant 15 jours ont été pris en charge par le service santé environnement d'une délégation territoriale départementale [de la région], et cela a été difficile à gérer, le service ayant atteint ses limites.	**Regional Health Agency (RHA), mainland France.** In 2023, one or two cases a day for 15 days were handled by the environment/health service of a departmental delegation [in the region], and this was difficult to manage, as the service had reached its limits.
D1-3	**Agence régionale de santé (ARS), France hexagonale.** En [région], la gestion de plusieurs cas autochtones sera très difficile à assurer au regard des moyens humains dédiés à ce jour surtout si l'activité des cas importés reste au niveau de 2023.	**Regional Health Agency (RHA), mainland France.** In [Region], it will be very difficult to manage several autochthonous cases, given the human resources currently available, especially if the activity of imported cases remains at the 2023 level.
D1-4	**Agence régionale de santé (ARS), France hexagonale.** La faiblesse des effectifs nécessite une certaine polyvalence des agents qui peuvent de fait être impactés par des demandes sur des sujets ne relevant pas de la gestion des arboviroses.	**Regional Health Agency (RHA), mainland France.** Our low staffing levels mean that our agents have to be versatile, and may be affected by requests on issues not related to arbovirosis management.
D1-5	**Agence régionale de santé (ARS), France hexagonale.** En fonction des épidémies d'arboviroses le nombre de cas et surtout le nombre de traitements va augmenter (réchauffement climatique/ zone d'implantation du MT [moustique tigre] en expansion) la gestion des signalements et de la mise en œuvre des traitements sera en augmentation alors que déjà les services sont en difficultés pour disposer de moyens humains relais. Les opérateurs risquent aussi d'être à un moment donné en difficultés, il est nécessaire qu'une solution d'appui dédiée à la métropole puisse être organisée et mobilisable par les ARS dès que leur opérateur est en difficulté pour réaliser les interventions.	**Regional Health Agency (RHA), mainland France.** Depending on arboviral epidemics, the number of cases and, above all, the number of treatments will increase (due to warmer weather/expanding *Ae. albopictus* zones). The management of reports and the implementation of treatments will increase at a time when services are already struggling to find the human resources they need. Operators are also likely to run into difficulties at some point, and it is essential that a support solution dedicated to metropolitan France can be organized and mobilized by RHAs as soon as their operator is in difficulty in carrying out interventions.
D1-6	**Opérateur de démoustication, France hexagonale.** Nous pouvons tenir sur ce rythme une quinzaine de jours en demandant un effort particulier de nos équipes. Cependant, si la situation dure, on pourra observer une fatigue de nos équipes et cela pourra affecter notre efficacité, nous serons obligé de réduire le nombre d'intervention en les espaçant.	**Vector Control Operator, mainland France.** We can keep up this pace for a fortnight if our teams make a special effort. However, if the situation lasts, our teams may become tired, which could affect our efficiency, and we may have to reduce the number of interventions by spacing them out.

Row number	Stakeholder & original response in French	Stakeholder & response translated in English
D1-7	**Agence régionale de santé (ARS), France hexagonale.** Volet communication grand public et mobilisation sociale à amplifier pour rendre acteur la population dans la lutte et adopter les gestes barrières adaptés :<br>• lors de retours de zones à risques<br>• lorsque la personne est malade<br>• par l'entourage du cas.	**Regional Health Agency (RHA), mainland France.** Amplify the communication with the general public and social mobilization to involve the population in the fight against the disease and adopt the appropriate barrier measures:<br>• when returning from high-risk areas<br>• when the person is ill<br>• by those around the case.
	**POPULATION HEALTH / HEALTHCARE**	
D2-1	**Médecin responsable d'un service dans un hôpital, DROM.** [Quels secteurs de l'offre de soins sont touchés par une épidémie de dengue / zika / chikungunya (médecine générale, urgences, hospitalisation, laboratoires, infirmiers...) ? De quelle façon ?] L'impact sur le système de santé est double : augmentation d'activité liée à l'augmentation du nombre de patients et diminution du nombre de professionnel de santé disponible car eux aussi touchés par l'épidémie. [Identifiez-vous des populations particulièrement vulnérables (d'un point de vue clinique) ?] Femmes enceintes, âges extrêmes, obésité, immunodépression, comorbidités + pour la dengue les syndromes drépanocytaires majeurs (SS, SC, Sβthalassémie). Ce dernier point est très important. La drépanocytose est le facteur majeur de risque de survenue de forme grave de de décès.	**A doctor working in a hospital, DROM.** [Which sectors of the healthcare system are affected by a dengue / Zika / chikungunya outbreak (general practice, emergency departments, hospital care, laboratories, nurses, etc.)? In what ways?] The impact on the healthcare system is twofold: an increase in activity due to the rising number of patients, and a reduction in the number of available healthcare professionals, as they are also affected by the epidemic. [Do you identify any particularly vulnerable populations (from a clinical perspective)?] Pregnant women, individuals at the extremes of age, people with obesity, immunosuppression, and comorbidities + for dengue, patients with severe sickle cell syndromes (SS, SC, Sβ-thalassemia) are at particularly high risk. This latter point is very important. Sickle cell disease is a major risk factor for severe forms and mortality.
D2-2	**Agence régionale de santé (ARS), DROM.** Planification pour : organiser des filières d'accueil et de prise en charge aux urgences.	**Regional Health Agency (RHA), DROM.** Planning for: organizing reception and care pathways in the emergency department.
D2-3	**Agence régionale de santé (ARS), DROM.** [...] mise en place d'une filière dengue dédié aux patient concernés par la dengue.	**Regional Health Agency (RHA), DROM.** [...] establishment of a dedicated dengue pathway for patients affected by dengue.
D2-4	**Agence régionale de santé (ARS), DROM.** Compte tenu de l'afflux aux urgences, il faudra organiser une filière spécifique et savoir ne retenir à l'hôpital que les cas sévères de dengue et les comorbides qui se sont compliqués avec la dengue.	**Regional Health Agency (RHA), DROM.** Given the influx to the emergency department, a specific care pathway will need to be organized, ensuring that only severe cases of dengue and comorbidities complicated by dengue are retained in the hospital.
	**ECONOMIC ACTIVITY / INFORMATION AND MISINFORMATION**	
D3-1	**Agence régionale de santé (ARS), France hexagonale.** L'impact en termes économiques est difficile à évaluer mais pourrait être comparable à niveau épidémique égal de celui observé lors des épidémies grippe/Covid. A noter toutefois un probable impact fort sur l'activité touristique lié à des déprogrammations de masse.	**Regional Health Agency (RHA), mainland France.** The economic impact is difficult to assess by could be comparable, at an equivalent epidemic intensity, to that observed during influenza or COVID epidemics. A potentially substantial impact on tourism resulting from mass cancellations.
D3-2	**Agence régionale de santé (ARS), France hexagonale.** La grande majorité des cas implique un arrêt de travail de 7 à 10 jours minimum, avec des personnes ayant une fièvre élevée pendant 3-4 jours et une asthénie persistante importante et incapacitante, non compatible avec une activité professionnelle. L'impact sur le monde de travail et sur les individus en cas d'épidémie se fera probablement sentir.	**Regional Health Agency (RHA), mainland France.** The vast majority of cases involve a work absence of at least 7 to 10 days, with patients experiencing high fever for 3–4 days and persistent, severe, and disabling fatigue, incompatible with professional activity. The impact on the workforce and on individuals in the event of an epidemic is likely to be significant.
D3-3	**Agence régionale de santé (ARS), DROM.** Compter entre 3 à 6 jours d'arrêt de travail par cas, sans compter les parents pour enfants malades. Donc un gros impact économique en cas d'épidémie massive.	**Regional Health Agency (RHA), DROM.** Between 3 and 6 workdays of absence per case, excluding parental leave to care for sick children. This would therefore imply a substantial economic impact in the event of a large-scale epidemic.

Row number	Stakeholder & original response in French	Stakeholder & response translated in English
D3-4	**Agence régionale de santé (ARS), DROM.** Impact touristique dans la région notable en cas de forte épidémie médiatisée mondialement.	**Regional Health Agency (RHA), DROM.** A major epidemic with global media coverage would have a significant negative impact on regional tourism.
D3-5	**Chambres de commerce et d'industrie (CCI).** Le secteur de la recherche et, par ricochet, celui de l'innovation seraient également sollicités. En effet, une épidémie entraîne la recherche de moyens efficaces pour y remédier. La création d'un vaccin, la recherche de nouveaux médicaments ou encore la création et mise sur le marché de nouveaux répulsifs efficaces mobiliseraient ce secteur [...] La mise en vente et commercialisation de nouveaux médicaments, de nouveaux répulsifs, ou de nouveaux vaccins sont des éléments qui peuvent également impacter économiquement l'industrie pharmaceutique.	**Chambers of Commerce and Industry (CCIs).** The research sector and, by extension, the innovation sector would also be mobilized. Indeed, an epidemic leads to the search for effective means to address it. The development of a vaccine, the discovery of new medicines, as well as the creation and market introduction of new effective repellents would mobilize this sector [...] The marketing and commercialization of new medicines, new repellents, or new vaccines are factors that may also have an economic impact on the pharmaceutical industry.
	**EDUCATION / RESEARCH**	
D4-1	**Agence régionale de santé (ARS), DROM.** [Parmi les acteurs les plus touchés, à partir de quel niveau de transmission (ou d'activité [...]) pensez-vous que les maladies transmises par les moustiques *Aedes* (dengue, Zika, chikungunya) auraient un impact significatif (de nature à modifier l'activité habituelle) sur d'autres acteurs : école par exemple (absence des enseignants/fermeture ponctuelle, autres) ?] Pas d'études ou d'éléments signalés sur ce sujet. À mettre en relation avec les jours de scolarité perdus ou les résultats obtenus en moyenne par les enfants, adolescents de cette région aux tests nationaux.	**Regional Health Agency (RHA), DROM.** [Among the most affected stakeholders, at what level of transmission (or activity [...]) do you think *Aedes*-borne diseases (dengue, Zika, chikungunya) would have a significant impact (sufficient to alter normal activities) on other stakeholders, such as schools (e.g., teacher absenteeism/school closures, etc.)?] No studies or elements reported on this subject. To be compared with the number of school days lost or the average performance of children and teenagers in this region on national assessments.
D4-2	**Agence régionale de santé (ARS), DROM.** Des cas d'absentéisme scolaire sont signalés à l'ARS pour suspicion de dengue y compris pour les enseignants mais pas de fermeture enregistrée sur l'épidémie en cours.	**Regional Health Agency (RHA), DROM.** Cases of school absenteeism are reported to the RHA on suspicion of dengue fever, including for teachers, but no closure is recorded for the current epidemic.
D4-3	**Agence régionale de santé (ARS), DROM.** Pour les écoles se posera la question de la gestion des absences des enseignants et de la désinsectisation des locaux de vie collective. Ces questions sont à anticiper.	**Regional Health Agency (RHA), DROM.** For schools, issues will arise regarding the management of teacher absences and the disinsection of shared facilities. These issues need to be anticipated.
	**SOCIETY**	
D5-1	**Agence régionale de santé (ARS), France hexagonale.** Concernant le traitement adulticide autour d'un cas / cas autochtone, nous avons régulièrement des personnes réticentes qui s'opposent ou font des réclamations, et ces situations sont toujours complexes à gérer. [...] La question récurrente de la communication ou non au grand public des zones concernées par les traitements / confidentialité : lorsqu'on a communiqué ces zones, nous avons eu des oppositions aux traitements (par des personnes n'habitant pas sur la zone). [...] De manière générale, les investigations de veille sanitaire se passent bien et elles sont l'occasion d'expliquer notre action et de donner des conseils de prévention. Les difficultés sont plutôt rencontrées lors de la mise en place de traitements adulticides.	**Regional Health Agency (RHA), mainland France.** Regarding the adulticide treatment around a case / autochthonous case, we regularly have people who are hesitant, oppose, or make complaints, and these situations are always complex to manage. [...] The recurring question of whether or not to communicate the areas affected by the treatments to the general public / confidentiality: when we communicated these areas, we faced opposition to the treatments (from people not living in the area). [...] Generally speaking, public health surveillance investigations go well and provide an opportunity to explain our actions and give prevention advice. Difficulties are more often encountered when implementing adulticidal treatments.

Row number	Stakeholder & original response in French	Stakeholder & response translated in English
D5-2	**Agence régionale de santé, France hexagonale.** Depuis 2022, en [Région], la population demande des interventions et la 'polémique' autour de l'usage d'insecticides n'est plus présente dans la population tant la nuisance peut être énorme [...] Plus souvent des craintes vis à vis du produit de traitement et de sa dangerosité. Le nombre de personnes réticentes augmente lorsque nous sommes amenés à faire plusieurs traitements (foyers de cas autochtones). [...] Des inquiétudes souvent remontées lorsque des ruches sont plus ou moins proches de la zone de traitement.	**Regional Health Agency (RHA), mainland France.** Since 2022, in [Region], the population has been requesting interventions, and the 'controversy' around the use of insecticides is no longer present, given the huge nuisance that can occur [...] More often, there are concerns regarding the treatment product and its potential dangers. The number of hesitant people increases when we need to carry out multiple treatments (outbreaks of autochthonous cases). [...] Concerns are often raised when beehives are more or less close to the treatment area.
D5-3	**Agence régionale de santé (ARS), DROM.** Les oppositions au traitement Dobol se caractérisent par un refus pur et simple du traitement proposé. Pas de médiatisation ou de militantisme développé, structuré contre cette activité, à cette heure.	**Regional Health Agency (RHA), DROM.** Opposition to the Dobol treatment is characterized by a straightforward rejection of the proposed treatment. There is currently no media coverage or organized activism opposing this activity.
D5-4	**Opérateur de démoustication, France hexagonale.** Réticences de certains habitants, gestionnaires et collectivités pour les contraintes d'accès (portails à laisser ouverts, clés/badges à prêter, horaires de passage). Réticence d'apiculteurs pour le risque d'impact (mortalité ou effets sublétaux) sur leurs colonies, ou parce que les délais pour déplacer les ruches sont estimés trop courts ou pour des raisons idéologiques.	**Vector control operator, mainland France.** Some residents, managers, and local authorities are hesitant due to access constraints (gates needing to be left open, keys/badges to be lent, passing times). Beekeepers are hesitant due to the risk of impact (mortality or sublethal effects) on their colonies, or because the time frame for relocating the hives is considered too short, or for ideological reasons.
D5-5	**Agence régionale de santé (ARS), DROM.** [Identifiez-vous des populations particulièrement vulnérables face aux arboviroses ?] D'une manière générale : les enfants et personnes âgées, les personnes atteintes de maladies chroniques, les femmes enceintes, les personnes drépanocytaires, les personnes en isolement social.	**Regional Health Agency (RHA), DROM.** [Do you identify any populations that are particularly vulnerable to arboviruses?] In general, children and the elderly, people with chronic illnesses, pregnant women, people with sickle cell disease, people in social isolation.

Table B.1: Excerpts from the responses provided by stakeholders to the questionnaires

3 SUPPL. C: EFFECTS BY TRANSMISSION INTENSITY LEVELS

The various effects we identified may manifest at different levels of transmission. Using our work,[3] we distinguished between five intensity levels for an autochthonous transmission episode, as reported in the Table below. Column (3) indicates the level at which we anticipated observing an effect in each specific domain.

(1)<br>Levels	(2)<br>Definitions	(3)<br>Domains
Level 0	"No autochthonous transmission, with or without the presence of imported cases, with or without the presence of tiger mosquitoes"	(No effect)
Level 1	"Episode of autochthonous transmission: presence of an autochthonous case or an outbreak (i.e., at least two cases clustered in time and space)"	Prevention, control, and management / Population health
Level 2	"Localized outbreaks: multiple episodes of autochthonous transmission, simultaneous or not, and without epidemiological links, or the presence of an outbreak with multiple transmission areas"	Prevention, control, and management / Population health / Healthcare / Economic activity / Research / Society / Information and misinformation
Level 3	"Epidemic: widespread distribution of autochthonous human cases beyond the already identified outbreaks"	Prevention, control, and management / Population health / Healthcare / Economic activity / Education / Research / Society / Information and misinformation
Level 4	"Major epidemic: an epidemic in an expanded area with a high incidence exceeding the surveillance capacities of previous levels, requiring adaptation of management measures and having multisectoral impacts"	Prevention, control, and management / Population health / Healthcare / Economic activity / Education / Research / Society / Information and misinformation

Source: ANSES (2024).

Table C.1: Definitions of intensity levels for an autochthonous transmission episode and effects on different domains by intensity levels (in mainland France)

4 SUPPL. D: INTERSECTORAL POLICY RECOMMENDATIONS

• *Develop an interministerial plan to combat outbreaks or epidemics and their effects.*

This would involve integrating both central and local actors into a comprehensive interministerial plan tailored to mainland France.

• *Strengthen and optimize vector control while preventing the overload of dedicated resources.*

This involves developing flexible and scalable strategies to manage surges in vector control demand, enhancing surveillance capacities, and providing targeted training to ensure that all relevant stakeholders can rapidly detect and respond to arboviral threats. Promoting risk awareness across the entire response chain is essential to avoid delays, enable early intervention, and reduce the risk of further spread. These efforts should be supported by reinforced international coordination, particularly during overlapping arboviral epidemics.

• *Encourage community engagement.*

Fostering public engagement in surveillance and control activities through citizen science and transparent communication is essential for effective epidemic preparedness.

• *Anticipate the emergence of commercial markets.*

Because an epidemic is likely to create opportunities for some economic actors (primarily in the fields of prevention and protection against risks), we recommend anticipating these opportunities and regulating the practices of the relevant stakeholders in these markets.

• *Support vulnerable population groups and ensure that inequality issues are addressed before epidemics.*

To reduce the risk of an epidemic exacerbating social inequalities, it is essential to implement proactive measures prior to its onset: ensuring equal access to protective measures, healthcare services, and information (related to arboviruses) across all segments of the population.

• *Promote and support research and innovation on arboviruses.*

We recommend improving knowledge by securing long-term funding for research on arbovirus dynamics, prevention methods, diagnostic tools, vaccine and antiviral treatments for arboviral diseases, and effects in multiple domains.

• *Foster international coordination.*

The global circulation of arboviruses directly influences the risk landscape in mainland France. Due to global travel, trade, and environmental changes, arboviral outbreaks abroad can rapidly impact domestic risk. We therefore emphasize the need for a more unified and coordinated global surveillance system, enabling early detection, real-time data sharing, and harmonized response strategies.

References

1 Nightingale A. A guide to systematic literature reviews. Surgery (Oxford). 2009 Sep;27(9):381-4.

2 Brown D. A review of the PubMed PICO tool: using evidence-based practice in health education. Health Promot Pract. 2020 Jul;21(4):496-8.

3 ANSES, Paty MC, Apouey B, Calba C, Cardinale É, Milcent C, et al. Epidémies dues à un arbovirus transmis par le moustique *Aedes albopictus* en France hexagonale: probabilité d'apparition, ampleur de la transmission et impacts sanitaires, économiques et sociaux. ANSES (French Agency for Food, Environmental and Occupational Health and Safety); 2024.
